# From Static Structures to Molecular Dynamics: Emerging Directions in X-Ray and Electron Materials Characterization

**DOI:** 10.3390/ma19173579

**Published:** 2026-08-23

**Authors:** Daisuke Sasaki, Kazuhiro Mio, Yuji C. Sasaki

**Affiliations:** 1 Graduate School of Frontier Sciences, The University of Tokyo, Kashiwa 277-8561, Japan; d.sasaki@edu.k.u-tokyo.ac.jp; 2 AIST-UTokyo Advanced Operando-Measurement Technology Open Innovation Laboratory (OPERANDO-OIL), National Institute of Advanced Industrial Science and Technology (AIST), Kashiwa 277-0882, Japan

**Keywords:** time-resolved measurement, diffracted X-ray tracking (DXT), diffracted X-ray blinking (DXB), gold nanocrystal probe, single-molecule measurement, transmission electron microscopy, large-volume data analysis, machine learning

## Abstract

Structural analysis using X-rays and electron beams has long provided the average arrangement of atoms and molecules—that is, “structural information”—with high precision. By contrast, static measurements cannot directly yield dynamic information on how a material changes over time; instead, information on motion is convolved into a single numerical value such as the B-factor (atomic displacement parameter). Taking this limitation as its starting point, this review surveys the recent trend of introducing a time axis into measurements to observe material dynamics directly. First, we outline the technological foundations that have made the transition from static to time-resolved measurement possible. It rests on the dramatic shortening of exposure times, enabled by the increased brilliance of X-ray and electron sources and by advances in detection technology such as direct photon-counting detectors. Next, we survey dynamic measurement techniques, including time-resolved X-ray crystallography, coherent X-ray scattering, neutron scattering, and time-resolved electron microscopy. We also point out the essential limitation that most of them still return ensemble or volume averages. Building on this, we systematically describe diffracted X-ray tracking (DXT), diffracted X-ray blinking (DXB), small-angle X-ray blinking (SAXB), transmitted X-ray blinking (TXB), and electron-beam molecular dynamics (EBMD), which use gold nanocrystals and gold nanoparticles as motion probes. We distinguish throughout between methods that follow individual objects—DXT and EBMD, which yield trajectories of single labeled molecules or single particles—and methods that analyze intensity fluctuations arising from many contributors within one pixel or illuminated volume—DXB, SAXB and TXB. The latter are not single-molecule measurements; rather, they replace a global ensemble average by a spatially localized statistical one, retaining local heterogeneity that a bulk measurement would average away. Finally, we discuss the implementation and prospects of the large-volume data analysis—principal component analysis, Bayesian inference, machine learning, and autonomous measurement—needed to handle the explosively increasing amount of information that the time axis introduces. We close with the outlook that time-resolved measurement incorporating AI and big-data analysis will become established as a new measurement platform that complements and extends conventional static structural analysis.

## 1. Introduction

The functions of materials are frequently expressed through the motion of atoms and molecules. Many of the phenomena that determine material properties originate not from the static average structure but from its temporal evolution. Examples include the dynamics of phase transitions, ionic conduction in solid electrolytes, relaxation of polymers, molecular rotation in catalysts, and the motion of filler particles.

However, X-ray crystallography and electron microscopy, the mainstream methods of materials characterization, principally yield time- and ensemble-averaged structures that are averaged both over time and over the molecular ensemble. Information on motion is condensed into a single indicator representing the “magnitude of fluctuation,” such as the B-factor (atomic displacement parameter) or the Debye–Waller factor. Yet this value contains contributions from both thermal vibration and static structural disorder, so separating the two from the numerical value alone is in principle difficult [1,2].

Introducing a time axis into the measurement is an effective way to overcome this limitation. Because time-resolved measurement can directly observe the motion of atoms and molecules themselves, it can provide insights that cannot be obtained from static structural information alone. For a long time, however, such measurements were constrained by the “exposure-time barrier.” The breakthrough came from the development of high-brilliance X-ray and electron sources, together with the advent of direct photon-counting detectors that almost entirely eliminate readout noise. These technological advances have drastically shortened exposure times, making continuous measurement with high temporal resolution possible.

On the other hand, introducing a time axis dramatically increases the volume of the acquired data and the amount of information they contain. Time-resolved, single-molecule, and operando measurements yield enormous data sets with multiple dimensions—time, space, and the number of molecules. Such data are difficult to analyze by human effort alone. Large-scale data analysis using dimensionality reduction, machine learning, Bayesian inference, and similar approaches therefore becomes indispensable, and this trend is closely related to recent developments in computer science.

**Figure 1:**
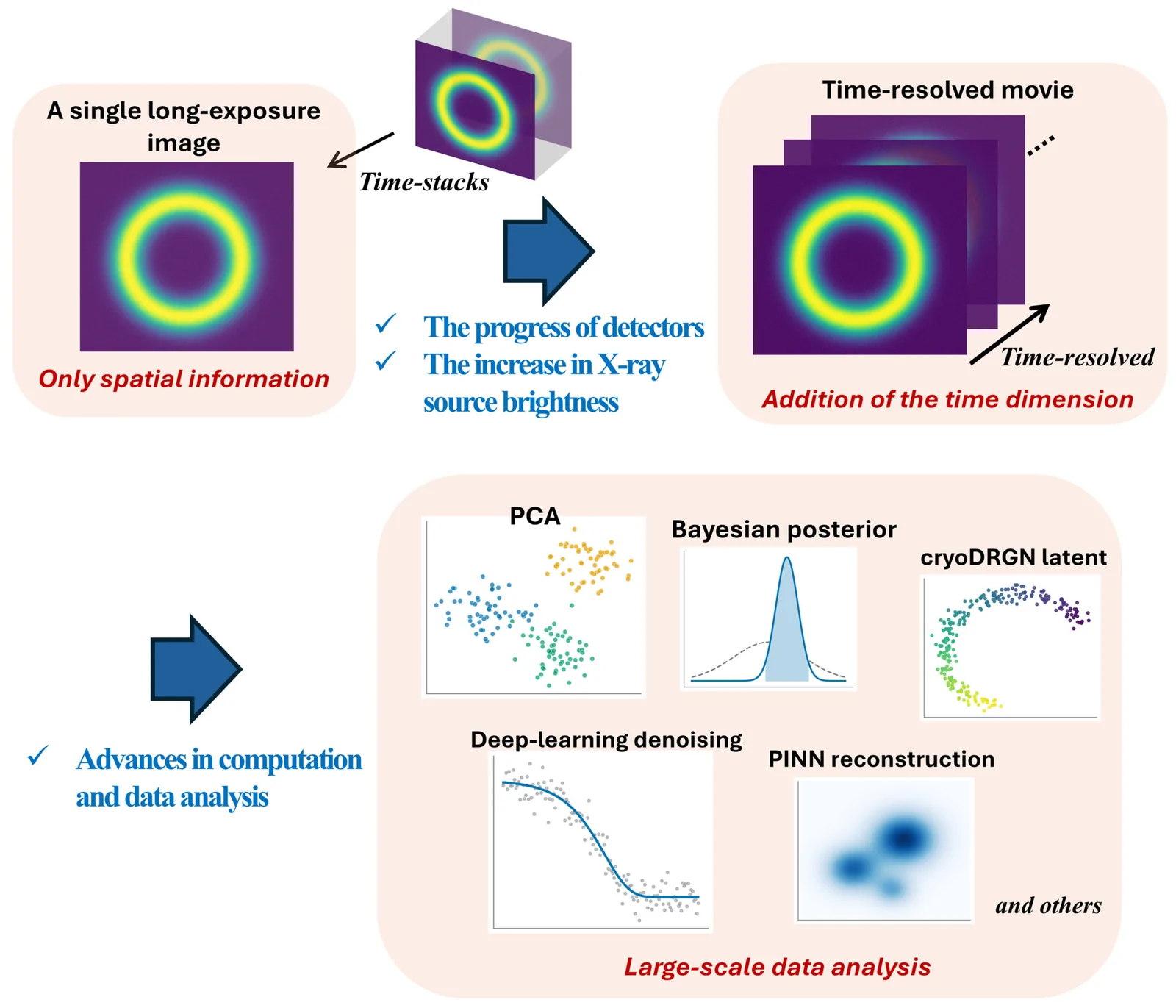
Conceptual overview of this review. A conventional measurement integrates over time and yields a single long-exposure image that carries only spatial (structural) information (left). Advances in detectors and in X-ray source brightness add a time dimension, turning the measurement into a time-resolved “movie” that captures how the structure evolves (right). The resulting large, high-dimensional data sets are interpreted using large-scale data analysis—principal component analysis (PCA), Bayesian inference, deep-learning denoising, physics-informed (PINN) reconstruction, and deep generative latent-space models (cryoDRGN), among others (bottom). Together, brighter sources, faster detectors, and advanced computation extend X-ray and electron characterization from static, ensemble/time-averaged structures toward single-molecule and single-particle dynamics. The panels in this figure are schematic illustrations and do not represent experimental data; the distributions shown are synthetic and are included for illustration only. They were rendered by plotting code written with the assistance of Claude Opus 4.8 (Anthropic PBC, San Francisco, CA, USA). No generative image model was used to produce any part of this figure.

In this review, we focus on three points. First, measurements that incorporate a time axis are advancing structural science from the analysis of static structures to the analysis of dynamic structures. Second, this change dramatically increases the amount of information that can be acquired, making advanced data analysis indispensable. Third, by incorporating information-science approaches such as AI and big-data analysis, time-resolved X-ray and electron-beam measurements are becoming a new foundation for materials characterization that complements and extends conventional static structural analysis (Figure 1).

Scope of this review. This review is written primarily for readers working on materials characterization. A substantial part of the single-molecule methodology discussed here was, however, first established using biological macromolecules, and we therefore use protein examples wherever they give the clearest demonstration of a measurement principle, indicating in each case how the same measurement transfers to non-biological materials. Neutron scattering is included only as a comparative benchmark for the time and length scales accessible to different probes and is not itself reviewed in detail; X-ray and electron probes remain the focus throughout.

The scientific contribution of this review is threefold. First, it identifies a common structure underlying a group of techniques that are normally presented in isolation from one another: in each, a nanoscale crystalline or high-contrast probe converts molecular motion into an intensity or angular fluctuation that a fast detector can record, so that an advance in one technique transfers directly to the others. Second, it establishes which of these techniques genuinely resolve individual objects and which return spatially localized statistics—a distinction that determines what may legitimately be concluded from the data, and one that is frequently blurred in the literature. Third, it argues that the limiting factor in this field has shifted from data acquisition to data interpretation, and that large-scale data analysis has consequently become part of the measurement itself rather than a step that follows it.

The review is organized as follows. Section 2 sets out what static structural analysis delivers and the advances in sources and detectors that made time-resolved measurement possible. Section 3 surveys the established dynamic measurement methods and identifies the averaging that most of them still entail. Section 4 describes the nanoprobe-based methods—DXT, DXB, SAXB, TXB and EBMD—that are the principal subjects of this review. Section 5 and Section 6 address two issues that cut across all of these methods: the perturbation introduced by the label and its attachment, and the effects of the probing beam itself. Section 7 turns to the large-scale data analysis required to interpret the resulting data volumes, and Section 8 summarizes the outlook.

This is a narrative rather than a systematic review. Literature was identified by searching Web of Science, Scopus, PubMed and Google Scholar for combinations of the keywords ‘time-resolved’, ‘X-ray’, ‘electron microscopy’, ‘single-molecule’, ‘diffracted X-ray tracking’, ‘diffracted X-ray blinking’, ‘X-ray photon correlation spectroscopy’, ‘machine learning’ and ‘autonomous experiment’, supplemented by the reference lists of the retrieved articles. The search covers publications up to June 2026. Because the aim is to trace a methodological trajectory rather than to quantify a body of evidence, no formal inclusion or exclusion protocol was applied; selection favored studies that establish a measurement principle or that demonstrate a decisive advance in time resolution.

## 2. Static Structural Analysis and Advances in Measurement Technology That Enabled Dynamic Measurements

Static structural analysis using X-rays has revealed the average structure, long-range order, and local coordination environment of materials with high precision. Single-crystal X-ray diffraction and powder X-ray diffraction (SC-XRD/PXRD) provide information on the average electron density and on the long-range order of crystals [3]. Small-angle X-ray scattering (SAXS) analyzes nanoscale structures and the morphology of soft matter. Fourth-generation synchrotron radiation sources have further improved the brilliance and spatial resolution of the X-rays available for SAXS measurements [4]. Pair distribution function analysis can characterize not only the average structure but also the local structure, including deviations from that average. However, the structures obtained by these methods are likewise averaged over a large number of atoms and molecules [5].

X-ray absorption spectroscopy (XAS), and in particular extended X-ray absorption fine structure (EXAFS), is sensitive to the local structure around a specific element, including the types, distances, and coordination numbers of the surrounding atoms. However, the Debye–Waller factor obtained from EXAFS analysis contains contributions from both the thermal vibration of atoms and the static disorder of the structure. Separating these two contributions from static measurements alone is therefore difficult [6]. This is a representative case in which atomic motion and static structural disorder are combined into a single value.

Electron microscopy is also one of the principal methods of static structural analysis. Through the so-called “resolution revolution,” single-particle cryogenic electron microscopy (cryo-EM) has made near-atomic-resolution structural analysis possible [7,8]. However, the three-dimensional structure is reconstructed by classifying and averaging a large number of particle images. The resulting structure therefore represents a representative structural state rather than a direct observation of the structural differences or temporal changes that exist within the same sample. Cryogenic electron tomography makes it possible to observe the internal structure of cells and materials in three dimensions in an environment close to that of the sample [9]. In conventional cryo-EM, however, the sample is measured in a frozen state, so the result is a static structure at a specific point in time.

X-ray diffraction and electron microscopy can thus reveal the positions of atoms and molecules and the variability of their structures with high precision. However, these static measurements alone make it difficult to determine directly how fast, in which direction, and over what range the atoms and molecules move. Quantities such as the B-factor in crystal structure analysis represent the variability of atomic positions, but their values may include contributions from thermal motion, static structural disorder, and analytical uncertainty. Atomic motion therefore cannot be extracted as temporal information from the B-factor alone [1]. To investigate the motion of atoms and molecules, the measurement must include a temporal axis.

It should be emphasized that static measurements are not devoid of information about motion. Atomic displacement parameters, multi-conformer and ensemble refinement, diffuse scattering, and three-dimensional classification in single-particle cryo-EM all recover disorder, heterogeneity and correlated displacement from time-averaged data. What a static measurement does not provide is a continuous trajectory: the time ordering of the states, and the rates connecting them, are lost. Conversely, many time-resolved methods reconstruct a sequence of ensemble-averaged states rather than observing a single object in motion. ‘Time-resolved’ and ‘single-molecule’ are therefore independent attributes of a measurement, and the methods discussed below differ in both.

The first factor that enabled the transition from static to dynamic measurement is the increased brilliance of X-ray sources. Delivering more X-ray photons to the sample within a short period of time yields a sufficient signal even with a short exposure time. The brilliance of synchrotron radiation sources has improved greatly as light-source technology has developed [10]. In recent years, fourth-generation synchrotron radiation sources adopting diffraction-limited storage rings (DLSRs) and multi-bend achromat (MBA) lattices have been constructed [11,12]. At the ESRF-EBS, a forerunner of these facilities, a brilliance improvement of about 100 times compared with conventional storage rings has been reported [13].

**Figure 2:**
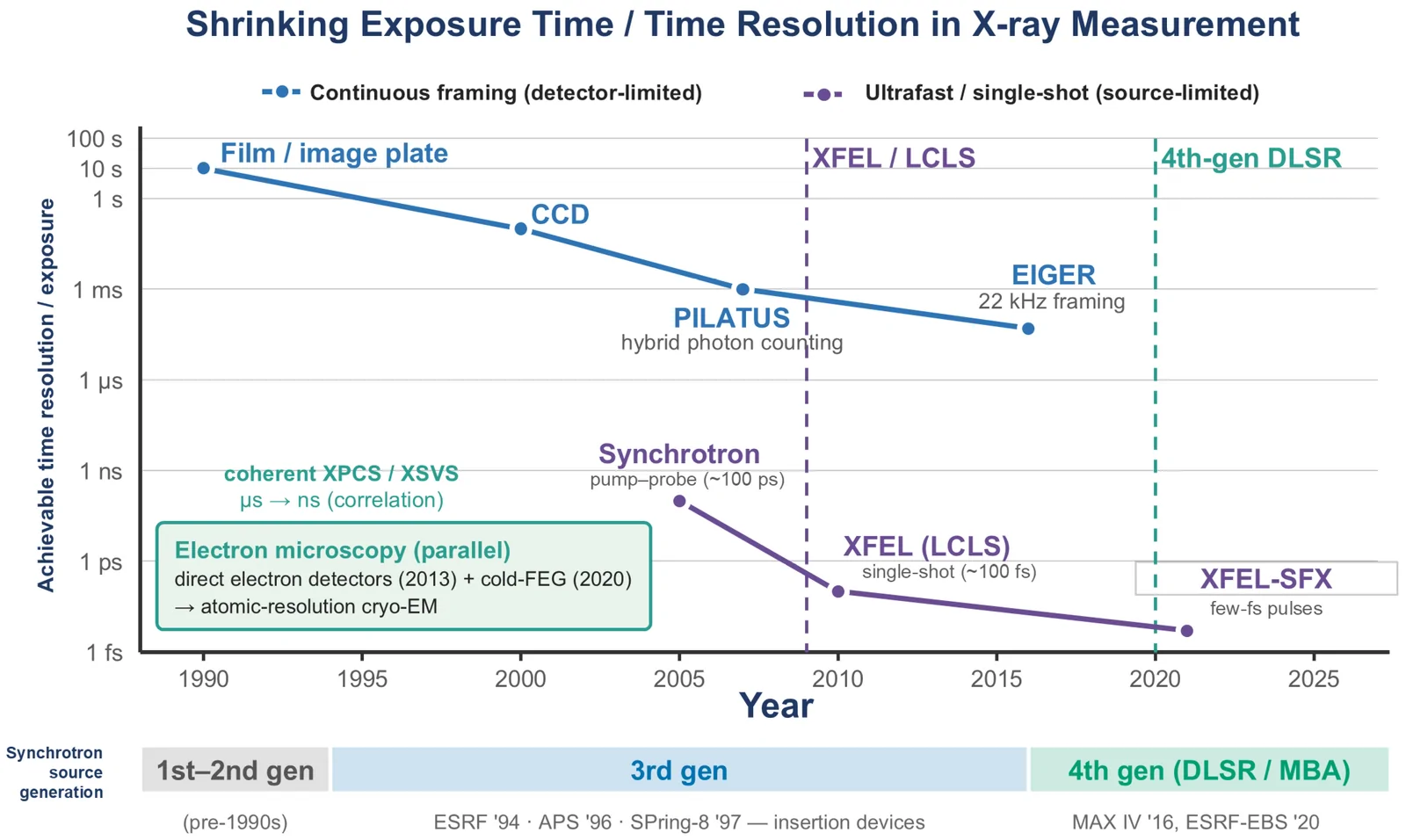
Shrinking exposure time/achievable time resolution in X-ray measurement over recent decades. Horizontal axis = representative year of introduction; vertical axis (log) = achievable time resolution/exposure. The quantities plotted here are related but not identical: some points represent an integrating exposure or a detector frame interval, others a source pulse duration, and others the demonstrated effective time resolution of a complete experiment. Blue: continuous-framing, detector-limited advances (film/image plate → CCD → PILATUS, hybrid photon counting → EIGER, 22 kHz framing). Purple: ultrafast, single-shot, source-limited advances (synchrotron pump–probe, ~100 ps → X-ray free-electron laser (XFEL)/LCLS single-shot, ~100 fs → XFEL-SFX, few-fs pulses). For pump–probe experiments, the effective resolution is set by the convolution of the pulse duration, the arrival-time jitter and the kinetics of reaction initiation, and is therefore generally poorer than the pulse duration alone. Parallel advances in electron microscopy (direct electron detectors, 2013; cold-FEG, 2020) enabled atomic-resolution cryogenic electron microscopy (cryo-EM). The band beneath the year axis marks the generations of synchrotron X-ray sources: 1st–2nd generation (pre-1990s; parasitic → dedicated rings), 3rd generation (from 1994; ESRF, APS, SPring-8; insertion-device-based, low-emittance), and 4th generation (from 2016; diffraction-limited storage rings with multi-bend achromat lattices—MAX IV 2016, ESRF-EBS 2020). Brighter sources and faster direct-counting detectors have together shortened the shortest accessible observation window by about 15 orders of magnitude, from the seconds of a conventional integrating exposure to the femtosecond pulse duration of an XFEL; note that these two end points are quantities of different kinds (see Table 1). The numerical value, the quantity represented and the source of every plotted point are listed in Table 1. Reference numbers follow the main text. This figure was rendered by plotting code written with the assistance of Claude Opus 4.8 (Anthropic PBC, San Francisco, CA, USA) and executed by the authors. All plotted values were taken by the authors from the cited literature and are listed, together with their sources, in Table 1. No generative image model was used to produce any part of this figure.

Synchrotron radiation sources have achieved these gains in brilliance over successive generations. The first generation used synchrotron radiation parasitically on accelerators built for high-energy physics experiments and was not dedicated to it. The second generation consisted of storage rings built primarily to use synchrotron radiation, and it relied mainly on radiation from bending magnets. In the third generation (ESRF, APS, SPring-8, and others from the 1990s onward), insertion devices such as undulators and wigglers became the main sources, and low-emittance electron beams greatly improved the brilliance and coherence. Fourth-generation synchrotron radiation sources adopting the DLSR/MBA lattices noted above advance this trend further by bringing the emittance of the electron beam close to the diffraction limit (Figure 2). The performance of the fourth-generation sources is now being documented from operation rather than from design: the emittance and coherence of the upgraded Advanced Photon Source have been measured directly [14], the first multi-bend achromat machine has been reviewed after several years of user operation [15], and the specific gains for time-resolved imaging have been set out explicitly [16].

**Table 1:** Numerical values plotted in Figure 2, with the quantity each value represents and its source. The synchrotron source-generation bands beneath the year axis, the coherent XPCS/XSVS annotation, and the electron-microscopy annotation (direct electron detectors, 2013 [17]; cold field emission gun, 2020 [18]) are described in the caption to Figure 2 and are not plotted as individual points.

Year	Development	Plotted Value	Quantity Represented	Ref.
1990	Photographic film/imaging plate	~10 s	Integrating exposure	[19]
2000	CCD detector	~0.1 s	Integrating exposure	[19]
2006	PILATUS hybrid photon-counting detector	~1 ms	Detector frame interval	[20,21]
2018	EIGER hybrid photon counting, 22 kHz continuous framing	~45 µs	Detector frame interval	[19,22,23]
2005	Synchrotron pump–probe crystallography	~100 ps	Effective time resolution	[24]
2009	LCLS: first lasing of a hard X-ray free-electron laser; single-shot exposure	~100 fs	X-ray pulse duration	[25]
2021	XFEL serial femtosecond crystallography	Few fs (<10 fs)	Effective time resolution (pulse-limited)	[24,26,27]

Furthermore, X-ray free-electron lasers (XFELs) can generate a much higher peak brilliance than synchrotron radiation, together with short X-ray pulses on the order of femtoseconds. This capability has made time-resolved measurements in the femtosecond regime possible [25]. The European XFEL can generate X-ray pulses at a high repetition rate on the MHz scale, so a large number of time-resolved data sets can be acquired in a short time [28]. The development of such new X-ray sources has expanded the temporal range over which phenomena can be observed even further [29].

The second factor that enabled dynamic measurements is the increased sensitivity and speed of detectors. In particular, direct photon-counting detectors, also known as hybrid pixel detectors, have greatly transformed X-ray measurement. PILATUS, a single-photon-counting detector, can detect individual X-ray photons and acquire images at high speed while remaining almost unaffected by readout noise [20,21]. Its successor, EIGER, achieves a frame rate of up to 22 kHz and a dead time of 4 µs [22,23].

These hybrid photon-counting detectors offer high sensitivity to single photons, low readout noise, a wide dynamic range, and high frame rates. Such performance has enabled continuous measurements without a mechanical shutter, diffraction measurements at fine angular intervals, and fast time-resolved measurements. Detector advances have raised the quality of diffraction and scattering data and have greatly improved the attainable temporal resolution [19]. Development of this detector family continues, with current-generation CdTe hybrid photon-counting detectors extending the approach to high photon energies [30].

Similar technological advances have taken place in electron microscopy. Electron sources have developed from thermionic guns to field emission guns (FEGs) and then to cold field emission guns (cold-FEGs), which can generate electron beams with higher brilliance and coherence. Combining a cold-FEG, an energy filter, and a direct electron detector has yielded a resolution of 1.7 Å in single-particle cryo-EM [18].

In particular, direct electron detectors, which can detect single electrons, greatly improved the signal-to-noise ratio of electron microscopy images and played an important role in raising the resolution of cryo-EM [7,17]. This development parallels the advances in direct photon-counting detectors in X-ray measurement. For both X-rays and electron beams, the concurrent development of high-brilliance sources and highly sensitive, high-speed detectors has made it possible to acquire high-quality data in a shorter time.

The increased brilliance of light sources and the greater speed and sensitivity of detectors have greatly shortened the exposure time required for X-ray measurement. Conventional static measurements sometimes required an exposure time on the order of seconds to minutes to acquire a single data set. At present, X-ray photon correlation spectroscopy (XPCS) is used not only for measurements in the millisecond regime but also for measurements in the microsecond regime [31]. Furthermore, split-pulse XPCS combined with XFELs can observe phenomena in the nanosecond regime [32], and single-pulse XFEL measurements can observe phenomena in the femtosecond regime [25]. In this way, the observable temporal range has expanded by about 15 orders of magnitude, from the seconds of a conventional integrating exposure to the femtosecond pulse duration of an XFEL (Figure 2). We stress that these two end points refer to quantities of different kinds—an integrating exposure time and a single-shot pulse duration—so that the figure of 15 orders describes the contraction of the shortest accessible observation window rather than the improvement of any one measurement modality. The like-for-like improvement in continuously framed time resolution, from film and imaging plates to megahertz-framing hybrid photon-counting detectors, is smaller by eight to nine orders of magnitude. The value plotted for each point in Figure 2, and the quantity it represents, are listed in Table 1.

Time-resolved crystal structure analysis can likewise now measure phenomena over a variety of temporal ranges. Pump–probe measurements using XFELs can observe reactions in the femtosecond regime, measurements using synchrotron radiation pulses reach the approximately 100-picosecond regime, and serial crystallography covers the microsecond-to-millisecond regime. Combining approaches such as cryo-trapping further allows reaction intermediates that proceed on time scales of seconds to minutes to be analyzed [24]. The temporal resolution actually attained is determined by the X-ray pulse width, the detector readout speed, the method used to initiate the reaction, the response time of the sample, and similar factors.

These shorter exposure times have made it possible not only to capture the structure of atoms and molecules as a static state but also to measure how that structure changes over time. High-brilliance sources and high-speed, highly sensitive detectors thus provide the technical foundation for the various time-resolved measurements described from Section 3 onward.

## 3. Modern Dynamic Analysis Methods

This section describes representative dynamic measurement methods that incorporate a time axis. Although these methods are collectively described as probing “dynamics,” the type of motion each one measures differs. The observables returned by the individual methods differ from one another, and the dynamics they capture correspond broadly to one of the following: (1) structural changes along a reaction coordinate induced by an external stimulus, (2) fluctuations and relaxation that arise spontaneously in equilibrium or steady states, (3) rotational and orientational motion of a single object, (4) translational diffusion of a single particle, and (5) the temporal evolution of strain or morphology. These methods therefore cannot be ranked by a single measure of temporal or spatial resolution alone; they can be compared only after clarifying what each method takes as its observable and which type of motion it measures. Each method is surveyed below from this perspective. In many of them, the analyzed signal originates from a large number of molecules, a large number of unit cells, or the entire region irradiated by X-rays or neutrons, so that directly tracking the motion of individual molecules or particles is difficult.

Serial femtosecond crystallography (SFX) uses an XFEL, and its time-resolved form (TR-SFX) observes the structural changes that accompany a reaction with high temporal resolution. The pump–probe method, in which the reaction is initiated by light, and the mix-and-inject method, in which the reaction is initiated by mixing the sample with a reactant, allow reaction processes ranging from femtoseconds to minutes to be analyzed [24,26]. Importantly, TR-SFX does not obtain a movie by continuously imaging a single crystal. Instead, it measures a large number of (often disposable) microcrystals while varying the delay time between the stimulus (pump) and the measurement (probe), and it reconstructs the time axis by integrating these measurements. The temporal evolution obtained is therefore a concatenation of the average structures of a trigger-synchronized molecular ensemble at each time point, not a trajectory that continuously follows a single molecule.

In one representative study, the structural changes accompanying the trans–cis photoisomerization of photoactive yellow protein (PYP) were observed with femtosecond temporal resolution [27]. Mix-and-inject serial crystallography has also been used to observe reaction intermediates of a riboswitch RNA [33]. These studies are important because they revealed, as a function of time, how atomic positions change as the reaction proceeds. However, TR-SFX determines structures by integrating diffraction data obtained from a large number of microcrystals, so the structures obtained represent the average state of the crystal ensemble at each time point. Recent work has addressed the practical limits of these experiments, including sample delivery by droplet microfluidics [34] and the quantification of optical scattering losses in pump–probe SFX [35].

Measurements using coherent X-rays are another important means of investigating the motion of materials. XPCS measures fluctuations and relaxation in equilibrium and non-equilibrium states by analyzing the temporal variation of the speckle pattern produced by the sample. This method can measure dynamics over a very wide time range, and the advent of fourth-generation synchrotron radiation sources and DLSRs is making it possible to observe faster and smaller motions [36,37]. The reach of XPCS has been extended by fourth-generation sources and XFELs: ultra-small-angle XPCS benefits directly from the Extremely Brilliant Source [38], and megahertz XPCS has now been demonstrated at the European XFEL [39].

X-ray speckle visibility spectroscopy (XSVS) analyzes the change in speckle contrast that occurs during a fixed exposure time, which makes it possible to evaluate motions faster than the frame rate of the detector. Split-pulse XPCS, which uses two X-ray pulses separated by a time delay, further enables the investigation of fast atomic and molecular dynamics that cannot be recorded directly with a conventional detector [40].

Bragg coherent diffractive imaging (BCDI) uses the coherent diffraction pattern obtained from a nanocrystal to reconstruct the internal shape and strain of the crystal in three dimensions. Applying this method in situ or under operating conditions makes it possible to track how the strain and defects inside a nanocrystal change over time [41]. BCDI can also target a single nanocrystal, but it does not track each individual atom or molecule within the crystal.

Neutron scattering is another important method for investigating motion within materials. Quasi-elastic neutron scattering (QENS) evaluates the diffusion, rotation, and local motion of atoms and molecules by analyzing the energy changes of scattered neutrons. Neutron spin echo (NSE) uses the spin of neutrons to measure small energy changes with high precision and thereby probes relatively slow relaxation motions. These methods are used mainly to analyze diffusion and relaxation in the time range of roughly picoseconds to nanoseconds [42,43]. Recent surveys set out the current state of NSE spectroscopy [44] and the application of QENS to ion mobility in energy materials [45].

In one example from materials science, lithium-ion diffusion in the solid electrolyte Li_10_GeP_2_S_12_ (LGPS) was measured by QENS [46]. The technique can provide information on the diffusion rate and the mode of motion of lithium ions. However, the measured scattering signal originates from the large number of atoms and ions present in the sample. It therefore does not directly track the migration path of a specific single lithium ion.

Time-resolved measurement is also advancing in electron microscopy. In time-resolved cryo-EM, the sample is rapidly frozen within a short time after it is mixed with a reactant. This procedure makes it possible to fix short-lived intermediates that exist at a specific time point after the initiation of the reaction and to observe their structures [47]. The sample is not imaged continuously during the reaction; instead, structures at different time points are compared by varying the time from reaction initiation to freezing.

For example, by mixing samples on a microfluidic chip and freezing them within about milliseconds, researchers have observed the intermediate states that appear in the HflX-mediated ribosome recycling process [48]. Although this method can capture short-lived structural states, reconstructing a three-dimensional structure usually requires classifying and averaging a large number of particle images. The structures obtained therefore represent the representative state of the particle ensemble present at each time point.

Time-resolved cryo-EM and time-resolved X-ray crystallography share a common concept: the reaction is initiated within a short time, and the structure is recorded after the targeted time has elapsed. The actual temporal resolution is set by how rapidly and uniformly the reaction can be initiated and by how quickly reaction intermediates can be frozen or measured. Advances in reaction-initiation and sample-preparation methods are making it possible to analyze the structures of reaction intermediates that arise on the millisecond-to-second timescale [49].

Time-resolved X-ray measurements, neutron scattering, and time-resolved electron microscopy are powerful methods for observing the structural changes that accompany reactions, as well as the fluctuations, diffusion, and relaxation within materials. However, the signals obtained in many of these methods contain contributions from a large number of molecules, particles, unit cells, or the entire irradiated region. Information on how individual molecules or particles move is therefore embedded within the averaged signal and is difficult to extract directly.

Moreover, even a method such as BCDI, which can observe a single nanocrystal, does not directly track the motion of individual molecules inside that nanocrystal. Measuring the motion of individual molecules or particles without relying on ensemble averaging requires that the signal obtained from each individual target be detected separately.

The methods described in this section are organized here according to the observable that each one returns and the type of motion it captures. TR-SFX and time-resolved cryo-EM take the average atomic arrangement or structure at each time point as their observable, and they capture structural changes along a reaction coordinate induced by an external stimulus. XPCS, XSVS, and split-pulse XPCS take the temporal autocorrelation of the speckle intensity as their observable, and they capture density fluctuations and relaxation that arise spontaneously in equilibrium and non-equilibrium states. QENS and NSE take the energy change of the scattering or the relaxation of the intermediate scattering function as their observable, and they capture spontaneous motions such as diffusion, rotation, and local motion. BCDI takes the strain field reconstructed from coherent diffraction as its observable, and it captures the temporal evolution of strain and morphology inside a nanocrystal. Even within the same class of dynamic measurements, then, the type of motion being measured differs from method to method. A difference in the observable changes the very object being compared, even when the time window or detection scale is similar. Beyond these methods, the approaches using gold nanocrystals and gold nanoparticles described in the next section capture still different observables that do not pass through ensemble averaging: the rotational, tilting, and twisting motion of a single molecule (DXT), the rotational and structural fluctuations evaluated from intensity fluctuations (DXB, SAXB, TXB), and the translational diffusion of a single particle (EBMD).

Finally, these methods also differ fundamentally in how they construct the time axis, and the physical meaning denoted by the word “dynamics” changes accordingly. First, in pump–probe-type methods such as TR-SFX and time-resolved cryo-EM, an external stimulus initiates the reaction, and the time axis is reconstructed by measuring a large number of (often fresh) samples while varying the delay time from the stimulus. The result is a kinetic trajectory in which the average structure of the synchronized ensemble advances along the reaction coordinate, rather than a continuous tracking of a single molecule (time-resolved BCDI measurements are likewise of the stroboscopic type, in that they repeat the drive and average over cycles). Second, methods such as XPCS, diffracted X-ray blinking (DXB), and QENS measure the correlation function of the equilibrium fluctuations that the system exhibits spontaneously, without applying an external stimulus. These measurements yield statistical quantities such as relaxation times and diffusion coefficients, not the trajectory of a specific single molecule. Third, diffracted X-ray tracking (DXT) and electron-beam molecular dynamics (EBMD) image a single target continuously in real time and obtain the stochastic trajectory itself. Even within the same class of time-resolved measurements, therefore, the dynamics being measured may be (i) the ensemble-averaged kinetics of an induced reaction, (ii) the relaxation time of spontaneous fluctuations, or (iii) the real-time trajectory of a single object. Which of these applies differs from method to method, so these measurements cannot be compared on a single scale.

## 4. Time-Resolved Dynamics Measurements of Single Molecules and Single Particles Using Gold Nanocrystals and Gold Nanoparticles

**Figure 3:**
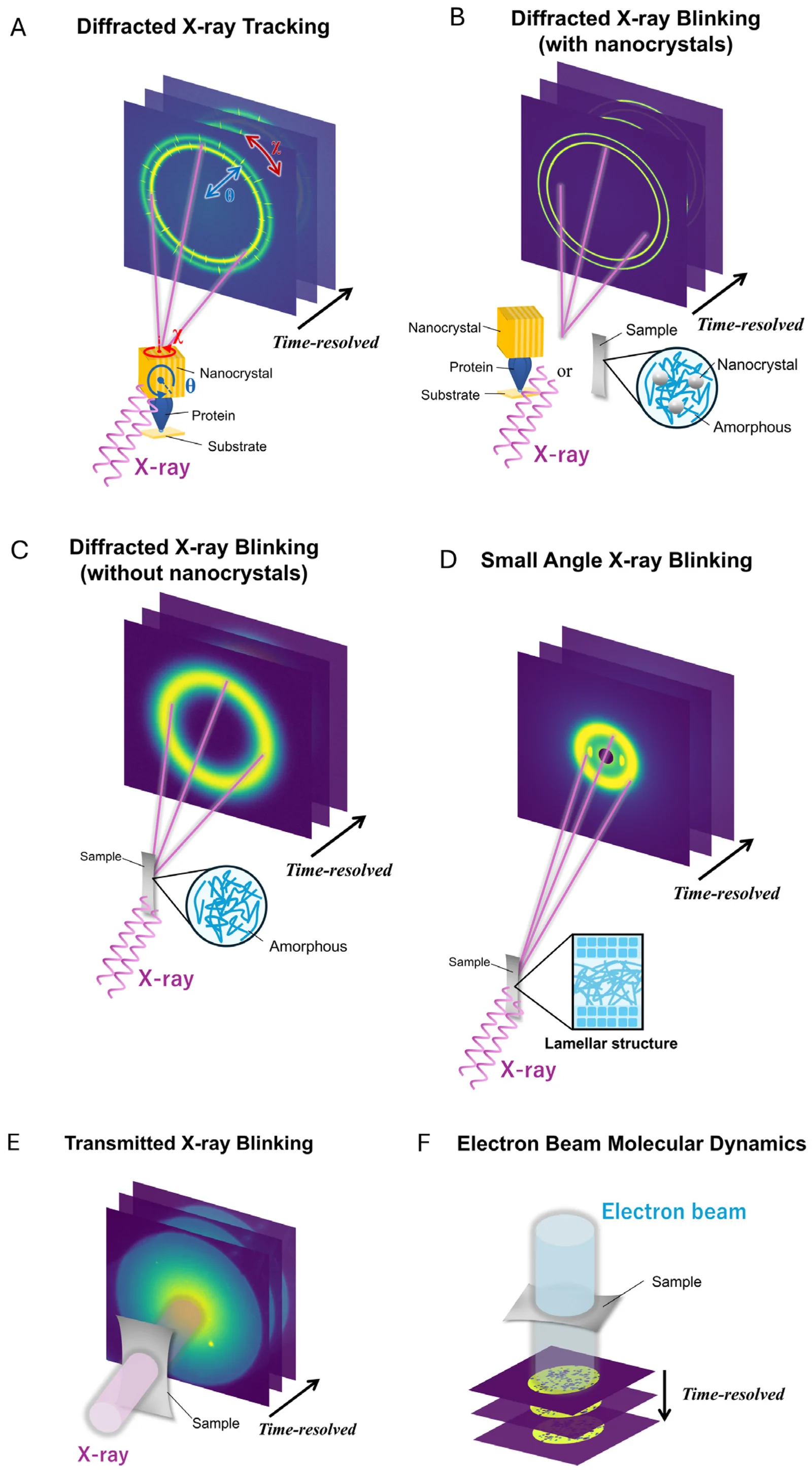
Schematic principles of the single-molecule/single-particle measurement methods discussed in this review. (**A**) Diffracted X-ray tracking (DXT): a gold nanocrystal conjugated to a substrate-anchored protein produces Laue diffraction spots whose tilting (θ) and twisting (χ) motions are tracked frame-by-frame. (**B**) Diffracted X-ray blinking (DXB) with nanocrystal labels: the diffraction-ring intensity from the labeled nanocrystals blinks as the crystalline domains rotate. (**C**) DXB without nanocrystal labels: the diffraction from crystalline domains within the (partially) amorphous material itself provides the blinking signal. (**D**) Small-angle X-ray blinking (SAXB): the small-angle scattering from a lamellar (crystalline/amorphous) structure blinks as the domain spacing fluctuates. (**E**) Transmitted X-ray blinking (TXB): the intensity of an X-ray image transmitted through a bulk material fluctuates (blinks) as gold nanoparticles or microcrystals dispersed inside the material move, and the local mobility is mapped pixel-by-pixel. (**F**) Electron-beam molecular dynamics (EBMD): an electron beam is used instead of X-rays to record the Brownian motion of gold nanoparticles on the sample in real space and in time-resolved form, from which per-particle trajectories are obtained. In (**A**–**E**), the time-resolved change of the diffraction, scattering or transmission pattern reports molecular or domain motion; in (**F**), the real-space particle position is followed directly.

This section describes time-resolved measurements that use gold nanocrystals or gold nanoparticles as motion probes (Figure 3). Analyzing changes in the diffraction spots or diffraction intensities produced by gold nanocrystals, or changes in the real-space position of gold nanoparticles, allows the motion of molecules and materials to be evaluated with high temporal resolution. The following sections treat, in order, DXT, DXB, small-angle X-ray blinking (SAXB), transmitted X-ray blinking (TXB), and EBMD. These five methods are compared in Table 2.

Gold is used for these probes for a combination of reasons. Its high atomic number (Z = 79) gives strong X-ray scattering and absorption contrast and strong contrast in transmission electron microscopy, so that a probe small enough not to dominate the system still produces a signal well above background. A gold nanocrystal can be grown as a single crystal with well-defined facets, so that the Au(111) reflection yields discrete Laue spots, or an intense and well-separated Debye–Scherrer ring, that can be used directly as an angular reporter. Because the diffraction from a single gold nanocrystal is intense, a usable signal is obtained within a very short exposure; this is what makes frame rates of microseconds—and, at an XFEL, of sub-microseconds—attainable at all, since a weaker scatterer would require integration times long enough to average out the motion of interest. Gold is chemically inert, stable in buffer and in air, and biocompatible, and thiol–gold and antibody-mediated conjugation chemistries are mature and site-specific. Finally, the particle size can be tuned from a few nanometres to about 100 nm, which allows the trade-off between signal strength and mechanical perturbation of the target to be adjusted deliberately. Where a second, spectrally distinguishable label is required, zinc oxide is used because its diffraction ring is well separated from that of gold, so that two labeled sites can be read out independently within a single measurement [70].

In DXT, white X-rays are irradiated onto a gold nanocrystal, or a gold-coated nanocrystal, bound to the target molecule. The motion of the Laue diffraction spots produced from that label is then tracked [50,51]. Following the position of a diffraction spot frame by frame measures the tilting motion of the molecule as θ and the twisting motion as χ. Because the diffraction spot produced from a single labeled gold nanocrystal is tracked individually, a characteristic feature of DXT is that it evaluates the rotational motion of the target molecule at the single-molecule level.

DXT detects minute angular changes with a temporal resolution on the order of microseconds. Quantities such as the mean-square angular displacement, calculated from the observed angular changes, further quantify the magnitude and directionality of molecular motion. The technique was initially developed mainly for biomolecules, but it has since also been applied to the dynamics analysis of materials and nanostructures [53].

DXT has been used to analyze the motion of various biomolecules. A study of tubulin showed that the neuronal and general-purpose isoforms differ in molecular mobility, and molecular dynamics (MD) simulations were used to examine the intramolecular structure responsible for this difference [71]. In the chaperonin CCT/TRiC, the loop motion of each subunit accompanying the ATP-driven protein-folding cycle was analyzed [72]. DXT has also been applied to soft materials, for example in the analysis of heterogeneity in hydrogels [58].

**Figure 4:**
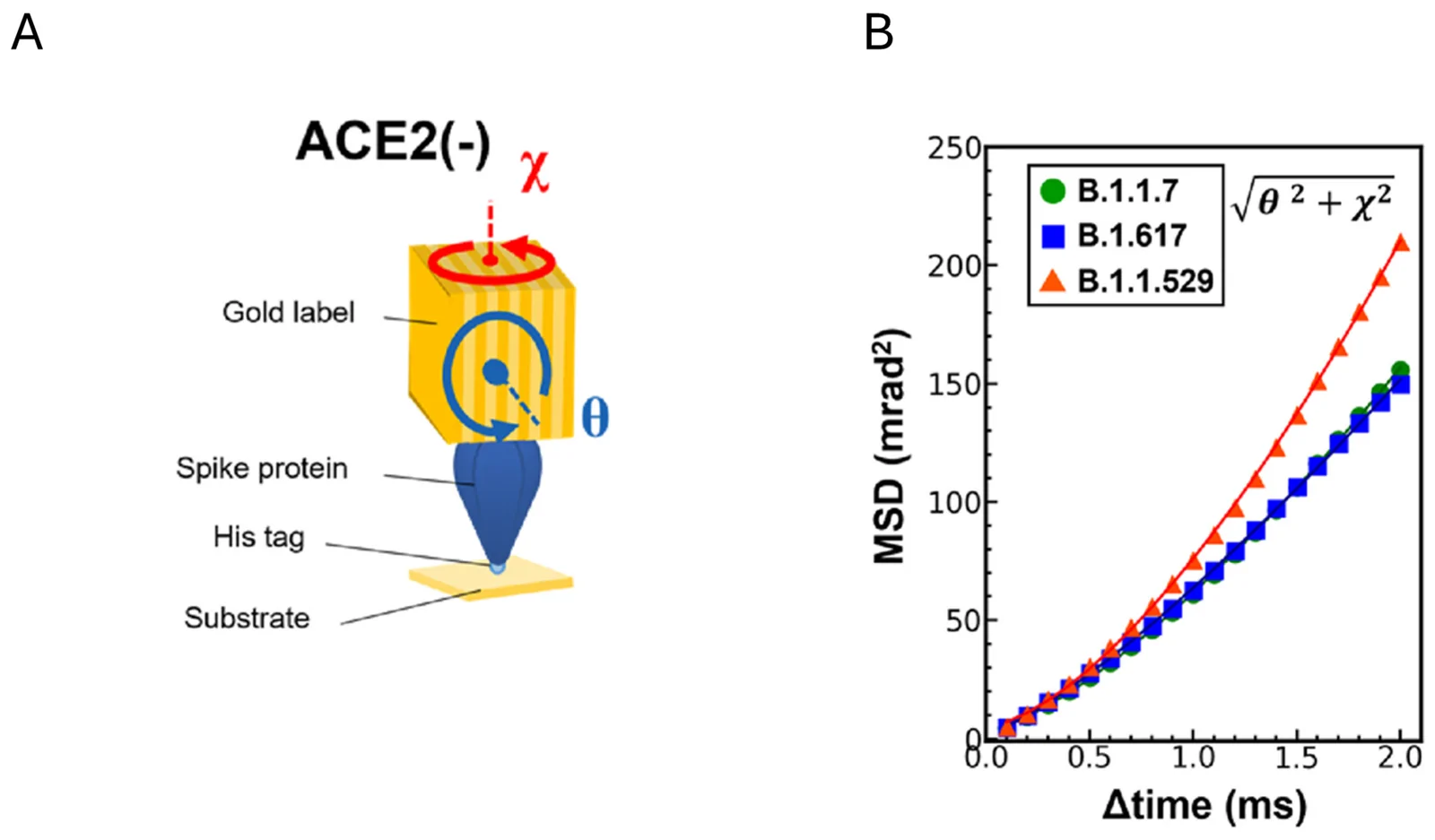
DXT of the intramolecular motion of SARS-CoV-2 spike-protein trimers in the receptor-free (ACE2(-)) state. (**A**) Measurement schematic: a His-tagged spike trimer is immobilized on a UV-hydrophilized polyimide substrate through Co^2+^ metal-affinity coordination, and a 60–80 nm gold nanocrystal is conjugated to a cysteine residue near the top of the trimer. The tilting (θ) and twisting (χ) rotations of the label are read out from the trajectories of the Laue diffraction spots at 100 µs per frame, reporting the internal motion of the protein. (**B**) Combined three-dimensional mean-square displacement √(θ^2^ + χ^2^) versus delay time (up to 20 frames = 2.0 ms) for the alpha (B.1.1.7, green), delta (B.1.617, blue) and omicron (B.1.1.529, orange) variants; the omicron variant shows the largest three-dimensional internal motion. Fitting each curve with r^2^(τ) = Dτ + b gives diffusion constants D of 58.5, 61.7 and 71.0 mrad^2^/ms for alpha, delta and omicron, respectively. Adapted from Ref. [52]; © 2024 The Authors; adapted with the permission of the copyright holders.

In the membrane protein TRPV1, the twisting motion of the intracellular domain was observed in real time to change upon capsaicin binding [56]. For the SARS-CoV-2 spike protein, the magnitude of internal motion has been shown to differ among variants (Figure 4) [52]. DXT can thus detect, at the single-molecule level, how the tilting and twisting motions of channels and receptors change upon events such as ligand binding [57].

**Figure 5:**
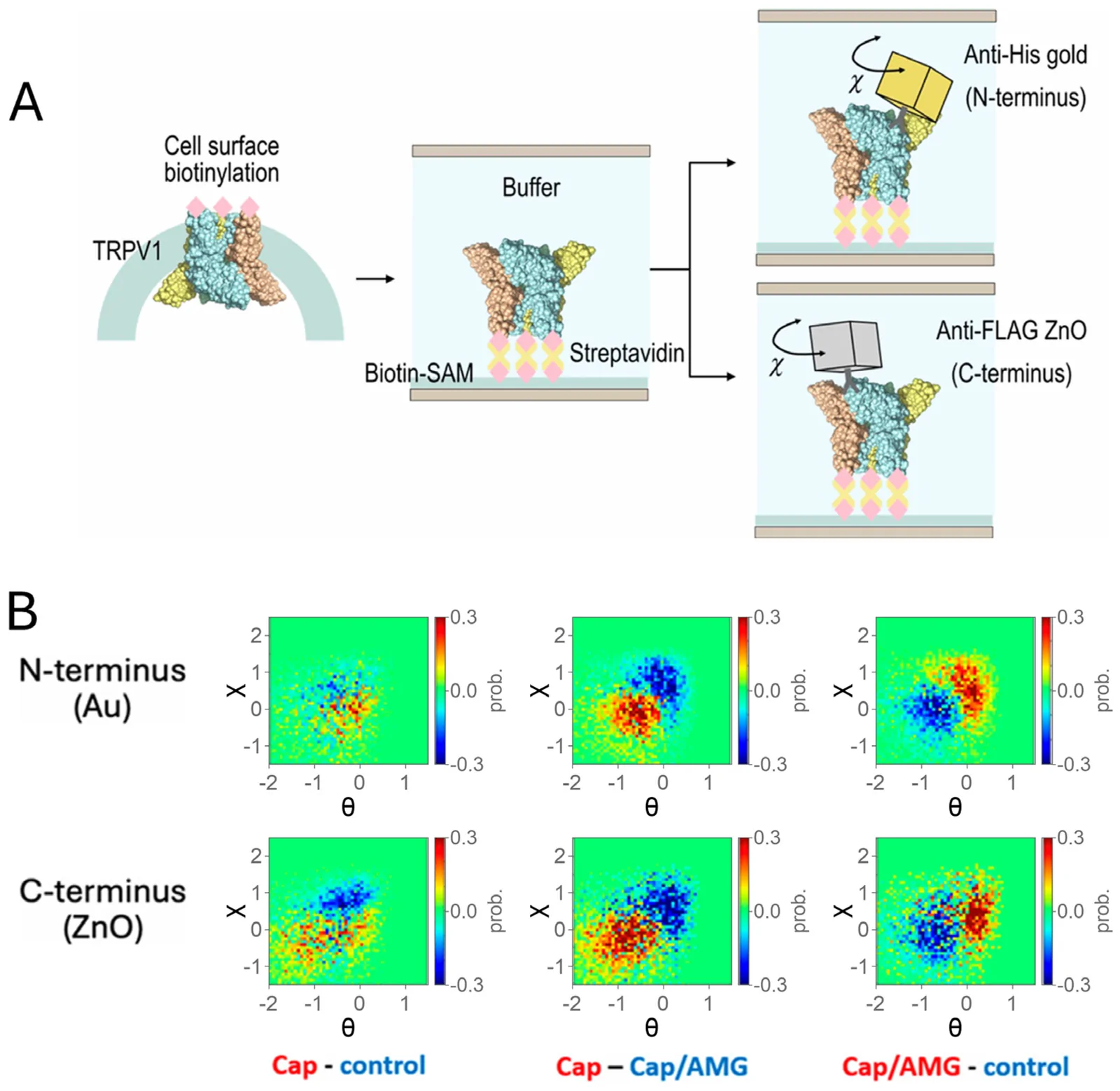
Simultaneous dual-labeled DXT measurement of the two cytoplasmic domains of TRPV1. (**A**) Labeling scheme: cell-surface-biotinylated TRPV1 is immobilized on a biotin self-assembled monolayer (biotin-SAM) through streptavidin, and its N-terminal His_6_-tag and C-terminal FLAG-tag are labeled with an anti-His gold (Au) nanocrystal and an anti-FLAG zinc-oxide (ZnO) nanocrystal, respectively, so that the motions of the two remote domains are recorded independently in buffer. (**B**) θ–χ probability-density difference maps for the N-terminus (Au) and the C-terminus (ZnO); the columns show the differences capsaicin − control, capsaicin − capsaicin/AMG9810, and capsaicin/AMG9810 − control (Cap = 10 µM capsaicin, agonist; AMG = 10 µM AMG9810, competitive antagonist). Capsaicin produced a clear peak shift and an overall change in motion at the N-terminus, whereas the C-terminus showed little positional change. Co-application of AMG9810 reversed the capsaicin-induced modulation, demonstrating that the two remote domains can be tracked independently under identical conditions. Adapted from Ref. [70] (CC BY 4.0).

Dual labeling with a zinc oxide nanocrystal and a gold nanocrystal has also been shown to allow simultaneous measurement of the motion of two separated sites within a single molecule [70]. This approach makes it possible to investigate how different sites within a molecule move in a coordinated manner (Figure 5).

Care is needed, however, regarding the relationship between DXT experimental results and MD simulations. In the tubulin study, for example, MD simulations were used to interpret the intramolecular mechanism that gives rise to the difference in mobility between isoforms. Directly calculating the angular changes obtained by DXT from MD trajectories and comparing them quantitatively with the experimental results has not yet been achieved. Connecting the diffraction-spot motion observed in experiments directly with the changes in atomic coordinates obtained by calculation requires a forward calculation that reproduces the observation process.

DXB uses monochromatic X-rays to measure the temporal variation of the diffraction intensity and evaluates crystal motion from its blinking [54]. When a microcrystal such as a gold nanocrystal rotates, it moves between states that satisfy the Bragg condition and states that do not. The diffraction intensity on the detector therefore increases and decreases over time. Analyzing this intensity variation with an autocorrelation function (ACF) or a similar treatment yields the motional velocity and relaxation time of the crystal.

Whereas DXT tracks the position of individual diffraction spots, DXB analyzes the temporal variation of the diffraction intensity recorded at each detector pixel. It is therefore easier to apply even under conditions where diffraction spots cannot be clearly tracked, and it can be carried out at a lower X-ray dose than DXT. DXB covers a wide time range from milliseconds to several thousand seconds, which makes it suitable for long-duration measurements and for measurements that suppress radiation damage.

**Figure 6:**
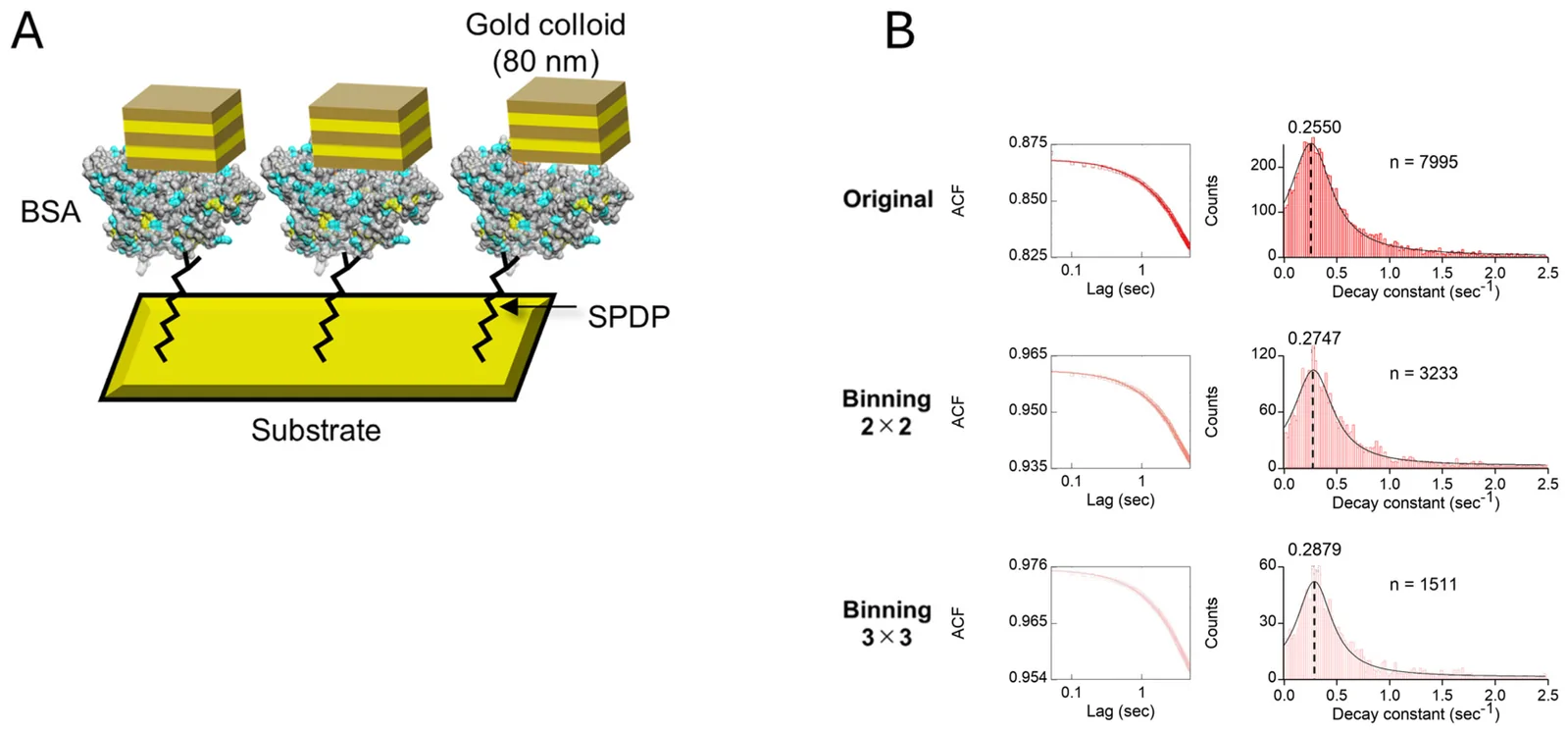
Laboratory-source DXB (lab-DXB) of bovine serum albumin (BSA). (**A**) BSA molecules immobilized on a gold substrate through the amine-to-sulfhydryl crosslinker SPDP and labeled with 80-nm gold colloids, whose Au(111) diffraction provides the blinking signal on the Debye–Scherrer ring under a laboratory Cu-anode X-ray source. (**B**) Averaged autocorrelation function (ACF) curves versus lag time (left) and the corresponding decay-constant histograms fitted with a Cauchy–Lorentz distribution (right) for three pixel-analysis conditions: Original (mode 0.2550 s^−1^, *n* = 7995), 2 × 2 binning (0.2747 s^−1^, *n* = 3233), and 3 × 3 binning (0.2879 s^−1^, *n* = 1511). The ACF decay constant increased with binning size, and the 3 × 3-binned data gave an estimated rotational diffusion coefficient of 0.73 pm^2^/s, showing that millisecond-order protein motion can be captured with a laboratory X-ray source. Adapted from Ref. [59] (CC BY 4.0). Following Ref. [59], the diffusion coefficient is obtained from the autocorrelation decay constant as D_R = ΓΦ_θ^2^/4, where Γ is the decay constant of the single-exponential fit ACF(t) = A exp(−Γt) + y and Φ_θ is the rotational displacement expressed as an arc length (Φ_θ = 3.18 pm for BSA at the Au(111) reflection). With Γ = 0.2879 s^−1^ for the 3 × 3-binned data this gives 0.73 pm^2^/s. Because Φ_θ is expressed as a length rather than as an angle, this quantity is a picometre-scale displacement diffusion coefficient associated with rotational motion and is not directly comparable with a conventional rotational diffusion constant expressed in rad^2^ s^−1^.

Lab-DXB, which uses a laboratory X-ray source, can measure the dynamics of protein crystals and polymer materials without access to a synchrotron radiation facility (Figure 6) [59]. XFEL-DXB, which uses an X-ray free-electron laser, instead observes motion in very short time ranges. In a study of a rubber material, for example, the dynamics of carbon black and polybutadiene were measured at a time interval of 890 ns [60]. GI-DXB, which uses a grazing-incidence configuration, evaluates molecular motion near the surface of polymer thin films with a laboratory X-ray source [61]. DXB has likewise been extended to molecular crystals, where it resolves polymorph-specific dynamics [62].

SAXB applies the blinking analysis used in DXB to the SAXS region. Whereas DXB mainly targets diffraction intensities originating from atomic arrangements, SAXB analyzes intensity variations in the low scattering-vector region. It can therefore evaluate structural fluctuations from the nanometer to the mesoscale, such as lamellar structures, domain structures, and aggregates.

**Figure 7:**
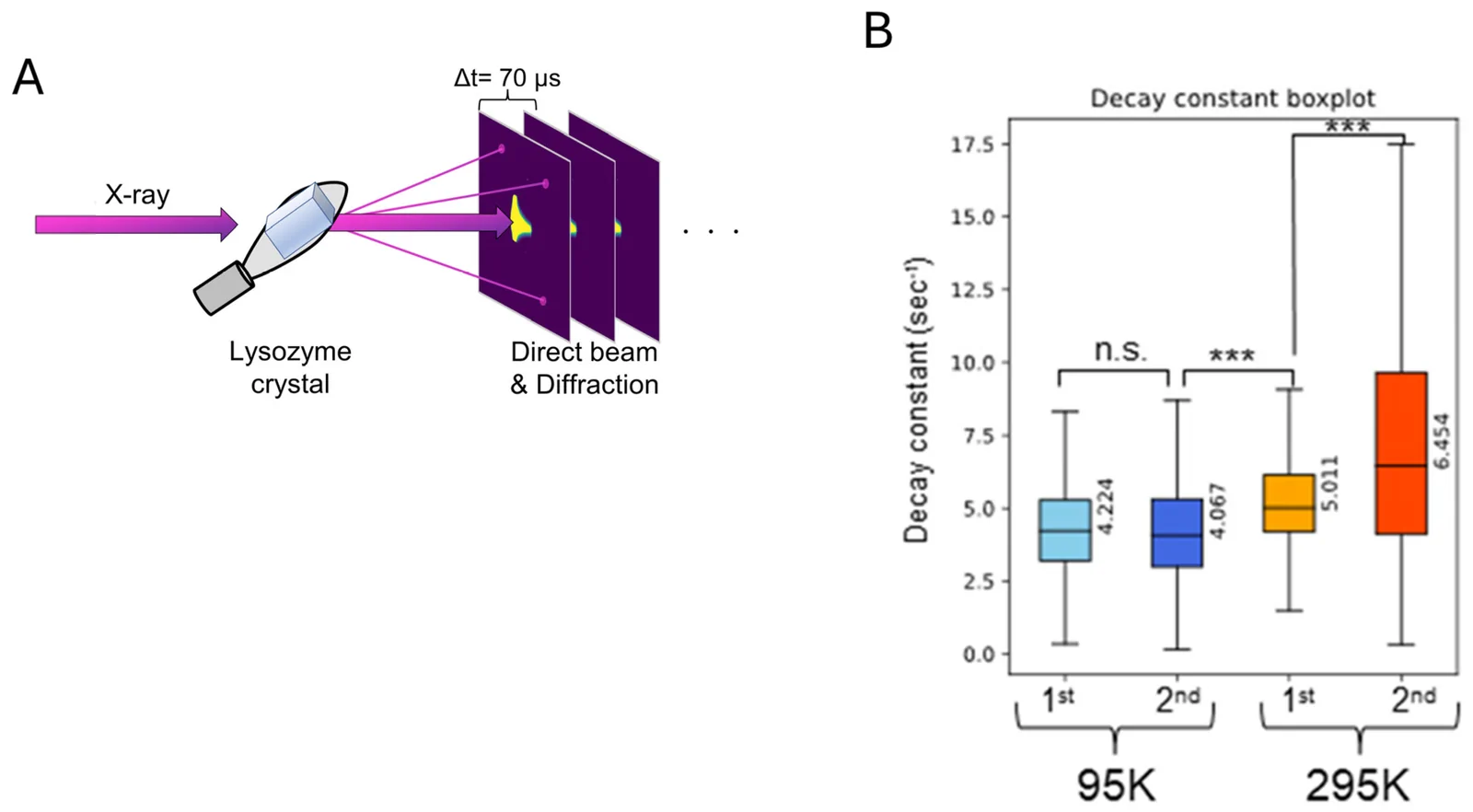
SAXB applied to a lysozyme single crystal. (**A**) Schematic: the X-ray direct beam and the diffraction from a lysozyme crystal are recorded with a 2D photon-counting detector at Δt = 70 µs, and the small-angle intensity fluctuation is analyzed pixel-by-pixel with an ACF fitted to a single exponential, ACF(τ) = A·exp(−Γτ) + y, whose decay constant Γ reflects the mobility of the crystal domains. (**B**) Boxplot of the 1-pixel ACF decay constants for two successive X-ray exposures at 95 K (median Γ = 4.224 and 4.067 s^−1^; n.s.) and at 295 K (5.011 and 6.454 s^−1^; significant, ***, *p* < 0.001, **, *p* < 0.01, *, *p* < 0.05, Wilcoxon rank-sum test). At 295 K, the domain mobility increases markedly upon repeated exposure, reflecting X-ray-induced crystal degradation, whereas at 95 K it is essentially unchanged. Adapted from Ref. [63] (CC BY 4.0).

SAXB determines the time scale of structural fluctuations by autocorrelation analysis of the temporal intensity variation recorded at each pixel of the small-angle scattering image. The method can evaluate the motion of nanostructures and the degradation process of protein crystals on short time scales, including the microsecond region (Figure 7) [63]. However, the scattering intensity observed in SAXB also includes contributions from many structures present within the irradiated volume. SAXB is therefore not a technique for individually tracking a single lamella or a single molecule, but rather one for statistically evaluating structural fluctuations at a specific spatial scale.

TXB analyzes intensity variations in X-ray images acquired in a transmission geometry. When X-rays pass through a bulk material such as a resin, the motion of gold nanoparticles or microcrystals dispersed within the material changes the transmitted and scattered intensities on the detector. Analyzing these temporal variations yields the local mobility inside the material.

**Figure 8:**
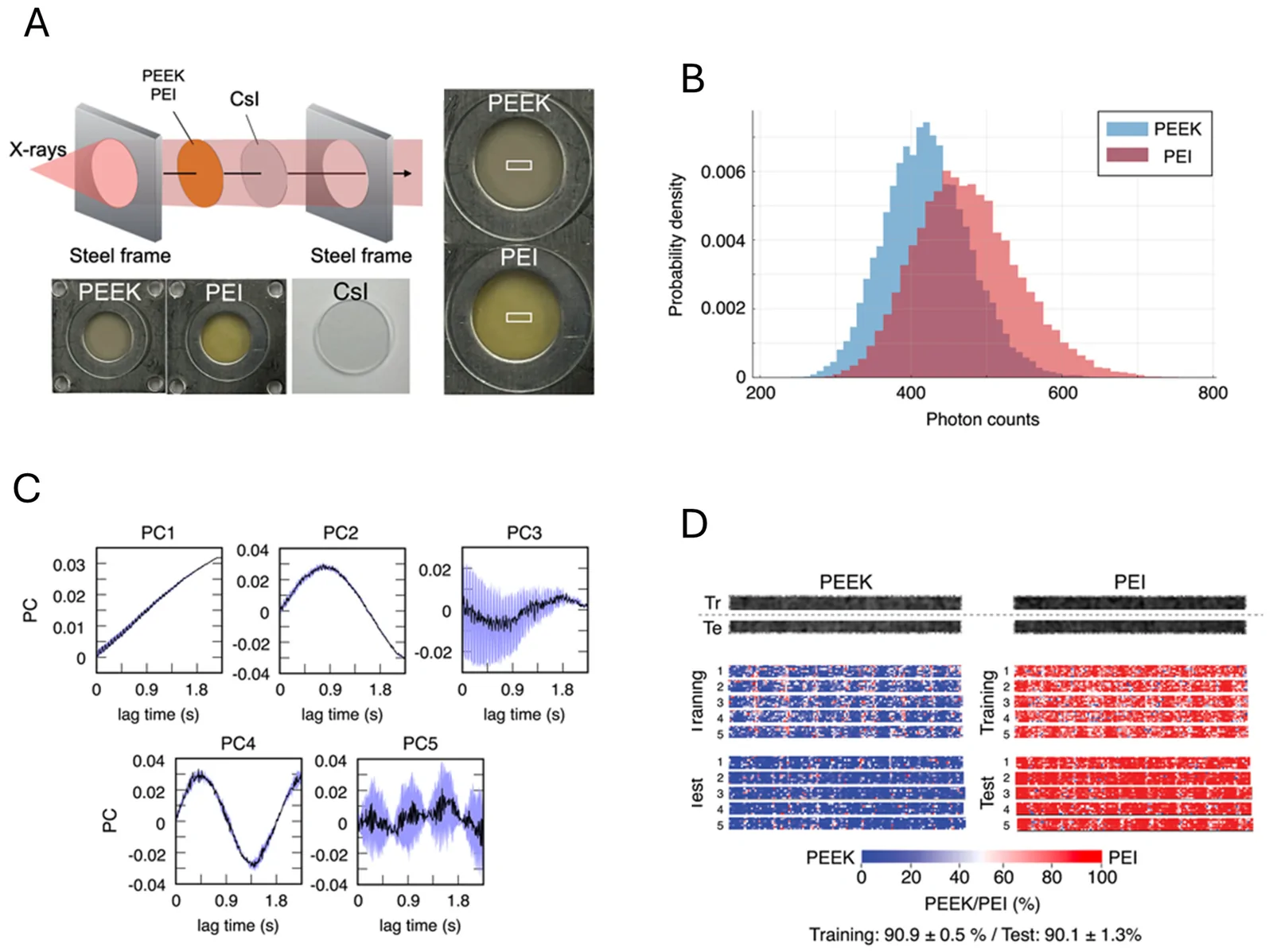
TXB analysis of semi-crystalline polyetheretherketone (PEEK) and amorphous polyetherimide (PEI), two resins with nearly identical X-ray absorption. (**A**) Benchtop transmission geometry in which a divergent laboratory X-ray beam passes through a polymer plate (10 × 10 × 1 mm) and a CsI scintillator held between steel frames; the emitted visible light is recorded by a high-speed camera at 900 ns per frame (~1.1 Mfps). Photographs show the PEEK, PEI and CsI specimens. (**B**) Probability density of the image-integrated photon counts for PEEK (blue) and PEI (red); the static transmission distributions overlap (absorption coefficients 0.208 ± 0.042 and 0.183 ± 0.038 mm^−1^), so absorption contrast alone cannot discriminate the two. (**C**) Representative first five principal components (PC1–PC5) from PCA of the single-pixel autocorrelation (spACF) curves. (**D**) Spatial maps of linear discriminant analysis (LDA) classification into PEEK (blue) and PEI (red) for the training and test sets, with accuracies of 90.9 ± 0.5% (training) and 90.1 ± 1.3% (test). Adapted from Ref. [64] (© Optica Publishing Group; Optica Open Access Publishing Agreement). The acquisition parameters are as follows. The value of 900 ns is the exposure time per frame, and this limit is set by the decay time of the CsI scintillator rather than by the camera. Each measurement comprises 10,000 frames recorded with a high-speed camera coupled through a tandem lens to a CsI scintillator of 10 × 10 × 1 mm. The single-pixel autocorrelation is computed on unbinned 900 ns samples, with no temporal binning, and is evaluated over 2500 lag points, giving a maximum lag of 2.25 ms. The frame grouping that appears in the principal-component score plots (250 segments of 10 frames each) is applied only to make the time series legible and is not part of the autocorrelation computation.

TXB applied to the engineering plastics polyether ether ketone (PEEK) and polyetherimide (PEI) visualized the mobility inside the materials two-dimensionally [64]. Analyzing the temporal variation of each pixel maps how the dynamics differ with position within the sample. Applying principal component analysis (PCA) and linear discriminant analysis (LDA) to the resulting time-series data was further shown to identify and classify different materials (Figure 8).

The concept of using gold nanoparticles as motion probes has been applied not only to X-ray measurements but also to transmission electron microscopy (TEM). EBMD is a method in which gold nanoparticles placed on a material surface or thin film are imaged continuously by TEM and their positional changes are tracked in real space.

EBMD determines the coordinates of each gold nanoparticle from consecutive images and analyzes their trajectories and mean-square displacement (MSD). The displacement, diffusion coefficient, and mode of motion of the particles can thereby be evaluated at the single-particle level. Treating the motion of the gold nanoparticles as a reflection of the mobility of the surrounding material allows phase transitions of the material and local molecular motions to be visualized.

For the synthetic polymers PC8FA and PSA, the motion of gold nanoparticles accompanying temperature changes was measured, and the phase transitions of the polymers were observed in real time [65]. Near the temperature at which melting or crystallization occurs, the mobility of the gold nanoparticles increased, and a peak appeared in the MSD. The result demonstrated that dynamic changes associated with polymer phase transitions can be detected by using the motion of gold nanoparticles present on the surface as an indicator.

**Figure 9:**
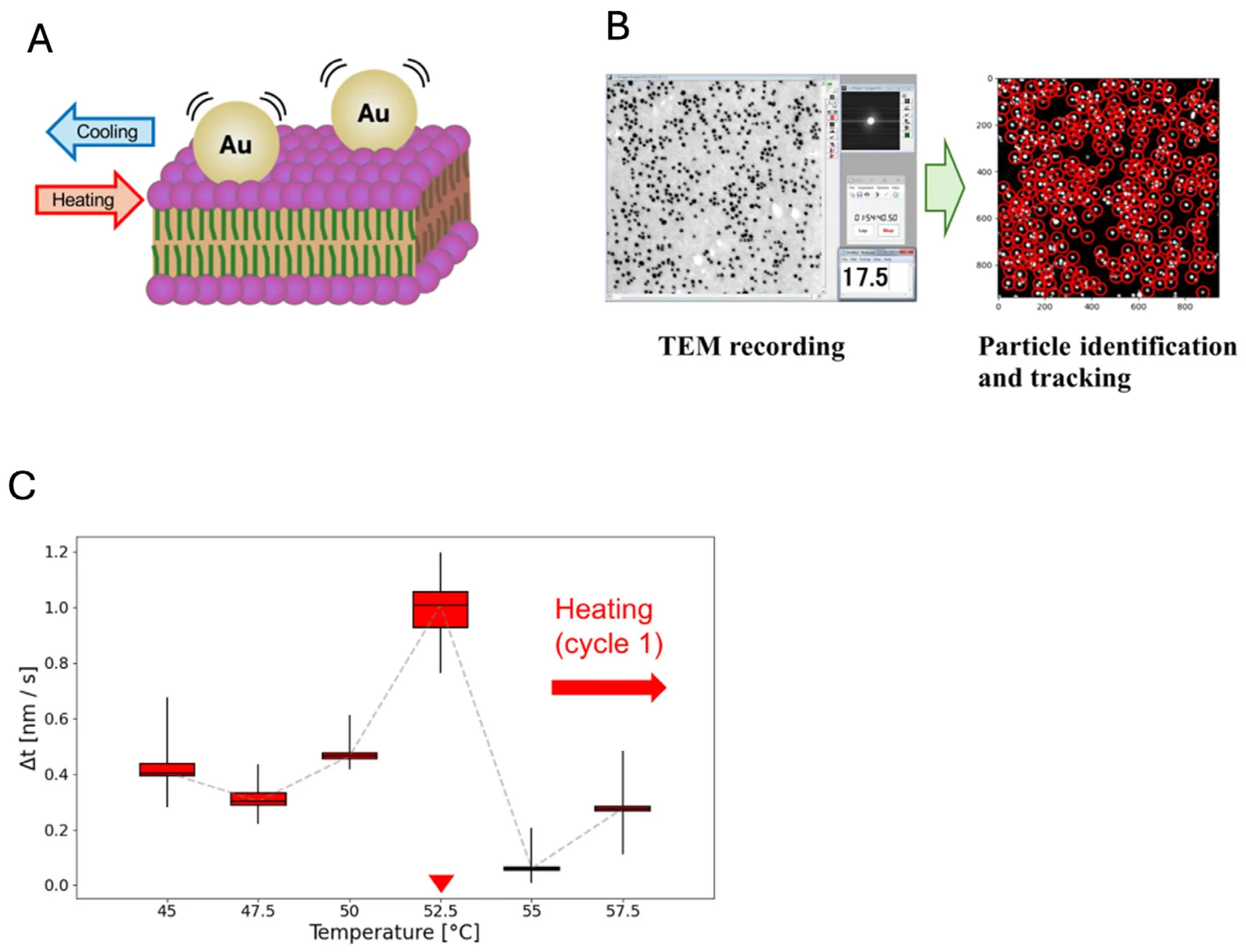
EBMD tracking of gold nanoparticles resolves the thermally induced phase transition of a DPPC lipid membrane. (**A**) Schematic of 5-nm gold nanoparticles dispersed as tracer probes on a lipid membrane spanning the ~2 µm holes of a Quantifoil grid, whose motion is followed by transmission electron microscopy (TEM) during controlled heating/cooling cycles. (**B**) Analysis workflow: real-space TEM videos are recorded, and individual nanoparticles are identified and linked frame-to-frame to build trajectories and mean-squared-displacement (MSD) profiles. (**C**) Box plots of DPPC nanoparticle mobility between 45 and 57.5 °C during the first heating cycle (box, 25th–75th percentiles; central line, median; whiskers, min–max), showing a pronounced peak in mobility near 52.5 °C that corresponds to the main gel-to-fluid phase transition of the dehydrated membrane. Adapted from Ref. [66] (CC BY 4.0).

EBMD has also been applied to the dynamic analysis of lipid membranes. In membranes composed of dipalmitoylphosphatidylcholine (DPPC) and dioleoylphosphatidylcholine (DOPC), changes in the motion of gold nanoparticles accompanying a rise in temperature were measured [66]. For DPPC, an MSD peak appeared near about 52.5 °C and was interpreted as a temporary increase in membrane mobility accompanying the main transition (Figure 9). DOPC, on the other hand, showed no distinct peak, but rather a continuous change in mobility with temperature.

EBMD complements ensemble-averaged measurements such as differential scanning calorimetry (DSC), fluorescence recovery after photobleaching (FRAP), and nuclear magnetic resonance (NMR). Whereas these conventional methods evaluate the average thermal properties and mobility of the sample as a whole, EBMD measures the trajectory of each individual gold nanoparticle in real space. It can therefore evaluate position-dependent differences within the same sample and the variability in mobility among individual particles.

Studies that track gold nanoparticles by electron microscopy are also advancing in systems that use liquid-cell TEM. Graphene liquid-cell TEM has been reported to show that gold nanoparticles of a few nanometers exhibit non-Gaussian diffusion that differs from simple Brownian motion. Rotational alignment mediated by ligands on the particle surface, together with the approach and coalescence of particles, was also observed in real space [67].

Deep-learning analysis of gold nanoparticle trajectories has further shown that the mode of diffusion changes with the electron dose. Fractional Brownian motion was observed under low-dose conditions, whereas motion closer to a continuous-time random walk was observed under high-dose conditions [69]. The electron beam itself may therefore influence the motion of the particles and the surrounding liquid environment. Dynamic measurements by liquid-cell TEM must accordingly evaluate the effects of electron dose and irradiation time with care.

Liquid-cell TEM is developing into a method that can observe the nucleation, growth, migration, rotation, and aggregation of nanoparticles in liquids in situ and in real time [68]. On the other hand, the thickness of the observable liquid layer, radiolysis caused by electron-beam irradiation, and interactions between the particles and the cell membrane may affect the measurement results. Examining the extent to which the observed particle motion reflects the intrinsic motion in the liquid is therefore important. Recent reviews summarize the rapid development of this field [73,74], and deep-learning pipelines now perform real-time instance segmentation of nanoparticles in liquid-phase in situ TEM [75].

The methods described above use gold nanocrystals and gold nanoparticles to obtain motional information that conventional static structural analysis could not provide. However, the information measured by each method differs. DXT evaluates the tilting and twisting motions of a single molecule by tracking a single diffraction spot. DXB and SAXB statistically evaluate the dynamics within the irradiated region from the temporal fluctuations of the diffracted and scattered intensities. TXB maps the mobility inside a material two-dimensionally, and EBMD tracks each individual gold nanoparticle in real space to determine its trajectory and MSD.

DXT and EBMD can thus track single molecules or single particles individually, whereas DXB, SAXB, and TXB are not necessarily methods that completely eliminate the ensemble average. Even so, analyzing the time-series data pixel by pixel on the detector makes it possible to evaluate local dynamics and spatial heterogeneity. Conventional approaches, which rely only on the average value of the sample as a whole, had difficulty capturing such local features.

The data obtained from these methods—angular changes of diffraction spots, intensity fluctuations, ACFs, MSDs, and particle trajectories—are very large. Relating the obtained values to molecular motion and material properties further requires statistical analysis, dimensionality reduction, machine learning, forward calculation, and inverse-problem analysis. The next section describes methods for analyzing these large-volume time-series data and extracting their physical meaning.

## 5. Influence of the Label, Linker, and Immobilization on the Measured Motion

In the methods described above, what is measured directly is the motion of the label, not of the target itself. The target is reported only insofar as the linkage between the two is stiff compared with the motion of interest, and with a gold nanocrystal of 60–80 nm attached to a protein a few nanometres across this cannot be assumed a priori. The size of the label, the compliance of the linker, the hydrodynamic load it imposes, and the immobilization of the target on a substrate all have the potential to alter the quantity being measured, and this deserves to be stated explicitly.

The studies cited above address this concern in four ways. The first is ligand specificity and reversibility: in the TRPV1 measurement, the change induced by capsaicin is reversed by the competitive antagonist AMG9810 [70], which an artefact originating in the label or the linker would not reproduce. The second is common-mode cancellation: the comparison among SARS-CoV-2 spike variants [52] uses identical labeling chemistry throughout, so that any contribution from the label is common to all variants and the differences between them remain interpretable even if the absolute values are not. The third is site specificity: dual labeling of the N- and C-terminal domains of the same molecule [70] gives different responses at the two sites, which cannot arise from a shared label artefact. The fourth is the use of substrate-only and unconjugated-label controls to establish the background level of apparent motion.

A further and more direct approach is to measure the size dependence within the measurement itself. Because the size of each gold nanocrystal can be read out from the normalized intensity of its own Laue diffraction spot, the relationship between label size and observed motion can be established across a single data set, without preparing separate samples. This was done for DXT of a sodium acetate solution, in which the angular displacement recorded over 25 µs was plotted against the normalized diffraction intensity of each label [55]. In the saturated solution the displacement depended on label size, as expected from the Einstein and Navier–Stokes relations, which predict a strong relationship between the size of a labeled particle and its dynamics; in the supersaturated solution it was largely independent of size, which was attributed to the nanocrystals being packed within ion-network domains. Extrapolating the fitted relation to zero diffraction intensity—that is, to a vanishing label—gave estimated label-free values of 87.1 and 27.5 mrad per 25 µs for the saturated and supersaturated conditions, respectively. That extrapolation is at present the most direct estimate available of what a DXT measurement would return in the absence of the label, and it illustrates the general point: the label-induced perturbation is not a fixed correction but depends on the system under study, being pronounced in a freely diffusing environment and small in a densely packed one. We suggest that this analysis be applied routinely wherever absolute angular velocities are reported.

The limitations that remain should nevertheless be borne in mind. Absolute amplitudes and absolute diffusion coefficients are affected by label size, linker compliance and surface immobilization, and immobilization removes global tumbling by design, so the motion observed is internal or local rather than whole-body. Comparative measurements made under identical labeling conditions are therefore considerably more reliable than absolute values, and the quantitative results quoted in this review should be read in that light. Establishing the magnitude of the label-induced perturbation directly—for example by systematically varying label size or linker length—remains an open experimental task.

## 6. Beam-Induced Effects and Their Influence on the Measured Dynamics

Because every method discussed here infers motion from a signal generated by an intense probing beam, the beam itself may alter the dynamics it is used to measure. The relevant quantities are the dose, the dose rate, the total irradiation time, and the resulting heating and radiolysis; these differ greatly among the methods and are worth comparing directly.

Among the X-ray methods, DXT carries the highest dose per frame, because it uses a white or pink beam and requires prolonged observation of the same field in order to build up trajectories. DXB at a synchrotron is intermediate, whereas laboratory-source DXB and TXB are at the opposite extreme: the flux of a laboratory Cu-anode source is lower by orders of magnitude, and radiation damage is correspondingly less of a concern, which is one of the practical attractions of these variants. SAXB provides an unusually direct measurement of the effect, since the same blinking signal that reports domain mobility also reports its change under irradiation: in a lysozyme crystal the decay constant increases significantly between two successive exposures at 295 K, while remaining unchanged at 95 K [63]. That is, the beam-induced contribution is itself observable in the data, and cryogenic measurement largely suppresses it.

On the electron side the situation is more severe. In EBMD and in liquid-cell TEM the dose rate is high, and radiolysis of water and of lipid, beam heating, and specimen charging can all contribute to the observed motion. The consequence must be stated plainly: part of the motion observed in these measurements may be beam-driven rather than thermally driven. That the diffusion mode of gold nanoparticles has been shown to change with electron dose is a direct demonstration of this concern rather than an incidental observation.

We therefore recommend that studies using these methods report the dose per frame and the cumulative dose, and that a dose series be used as a routine control to establish that the measured dynamics are dose-independent over the range employed. Where a dose-dependence is found, the extrapolation to zero dose—rather than the value measured at the working dose—is the quantity of physical interest.

## 7. Implementation of Large-Scale Data Analysis and Future Prospects

Time-resolved measurements, single-molecule and single-particle measurements, and operando measurements all acquire data with multiple dimensions such as time, space, and particle number. Faster detectors with higher pixel counts have further increased the volume of data acquired. Analyzing individual images and trajectories by hand is therefore difficult, and automated large-scale data analysis becomes necessary. In this section, we describe representative analysis methods used in dynamics measurements, together with future prospects.

Throughout this section we distinguish explicitly between approaches that have already been demonstrated for the techniques reviewed here and approaches that remain prospective. Several of the methods discussed below are well established for XPCS, small-angle scattering or single-particle cryo-EM but have not yet been applied to DXT or DXB; where that is the case we say so, because the distinction determines whether a reader may treat the approach as available or as a research direction.

PCA is a representative method for extracting the principal features of high-dimensional data. In MD simulations, PCA is widely applied to the temporal variation of atomic coordinates to extract the principal directions of molecular motion, an approach known as essential dynamics [76]. Transforming the complex motions of numerous atoms into a small number of principal components makes the large-scale motions common to the entire molecule easier to understand.

PCA has also been applied to the dynamics measurements treated in this review. In a study that applied TXB to PEEK and PEI [64], PCA reduced the dimensionality of the time-series data obtained from each pixel. LDA was then used to identify and classify the materials. PCA extracts the principal components that represent the variability of the data, whereas LDA finds the axes that best discriminate among known material classes. This combination makes it possible to extract material-specific features from large amounts of time-series data.

Machine learning is also used to analyze large amounts of scattering and diffraction data. Applying PCA to diffuse scattering data has been shown theoretically to extract a small number of features that represent the correlated disorder of atoms [77]. Interpretable unsupervised machine learning has also been reported to enable the detection of phase transitions and changes in order parameters from large amounts of X-ray diffraction data, without the analyst providing classification criteria in advance [78].

Dimensionality reduction of this kind is effective for making large amounts of data more tractable. However, the extracted principal components do not necessarily correspond to a single, well-defined physical motion. Because the principal components represent directions of large variance in the data, they may also capture instrumental drift, changes in sample position, and fluctuations in irradiation intensity. Judging their physical meaning therefore requires comparison with the experimental conditions and known material properties.

Estimating structure and motion from experimental data is generally treated as an inverse problem. In an inverse problem, multiple structural or motional models may explain a single observation. A unique answer is therefore difficult to determine from the observed data alone. Bayesian inference is a powerful method for treating such inverse problems involving uncertainty.

Bayesian inference combines prior knowledge about structure and motion with experimental data and evaluates the plausibility of each model as a probability. In the Bayesian ensemble reweighting method (BioEn), for example, the weights of a structural ensemble obtained by simulation are adjusted to estimate a structural distribution consistent with the experimental data [79]. A distinctive feature of this approach is that, rather than seeking a single optimal structure, it yields a probability distribution over multiple structures.

Methods for integrating experiment and simulation have been widely studied in structural biology and materials science [80]. MD simulations provide atomic-level trajectories, but their results depend on computation time, force fields, initial structures, and similar factors. Experimental data, in contrast, reflect the actual sample but provide only limited observable information. Combining the two compensates for the weaknesses of each and makes it possible to build a dynamics model consistent with the experimental data.

Simulation-based inference (SBI) has also attracted attention in recent years [81]. SBI generates a large amount of simulated data from a physical model and estimates the probability distribution of the model parameters by comparing them with the experimental data. One advantage is that SBI can be applied even to complex systems for which the likelihood function is difficult to write down explicitly. Combined with posterior estimation using neural networks, it can evaluate a large number of candidate models rapidly.

Deep learning is also used to estimate structure from scattering data. A method has been reported for estimating a three-dimensional shape model from the one-dimensional profile of solution SAXS [82]. Conventional SAXS analysis often compares experimental data with a shape model or structural model assumed in advance. Deep learning instead learns the relationship between a large number of structures and their scattering profiles, allowing shape candidates to be estimated rapidly from experimental data. Related approaches include machine-learning inversion of small-angle scattering from charged polymers [83] and neural-network recovery of reaction mechanisms from time-resolved crystallographic data [84].

However, determining a three-dimensional structure uniquely from a one-dimensional SAXS profile is fundamentally difficult, because different three-dimensional structures can produce similar scattering profiles. Estimates obtained by deep learning should therefore not be treated as the unique structural solution. They must instead be evaluated together with information from electron microscopy, crystal structure analysis, molecular simulation, and related approaches.

When inverse problems are solved with machine learning, physics-informed machine learning, which incorporates physical laws into the learning process, is effective. In physics-informed neural networks (PINNs), imposing the governing equations and boundary conditions as part of the loss function makes it easier to obtain physically consistent solutions even from a small amount of noisy observational data [85]. Compared with purely data-driven learning, this constraint guides the search toward physically plausible solutions, which offers advantages in extrapolation beyond the range of the training data and in the suppression of non-physical solutions [86]. Attempts to incorporate a differentiable forward model of diffraction into a neural network and reconstruct structure from coherent X-ray diffraction patterns have been reported [87], and it is considered that similar physics-constrained inverse analysis could also be applied to DXT and DXB. It should be noted that, to our knowledge, PINNs have not yet been applied to X-ray or electron scattering data of the kind discussed in this review; the demonstrations to date lie in adjacent inverse-problem domains [88]. The discussion here is therefore prospective rather than a description of established practice.

In inverse problems, multiple solutions can explain the observed data to a comparable degree, so quantifying the uncertainty of the estimation results is important. Representative methods for attaching uncertainty to deep-learning predictions include deep ensembles, which integrate the predictions of multiple models [89], and Bayesian deep learning, which separates data-derived variability (aleatoric uncertainty) from model-derived uncertainty (epistemic uncertainty) [90]. Such methods make it possible to distinguish regions where the estimation is reliable from regions where the data are insufficient and the estimation is unreliable [91]. Combining them with the evaluation of identifiability described in the previous section makes it possible to quantify how reliably molecular motion can be estimated from observed data.

Machine learning and deep learning are also used to analyze intensity fluctuations and correlation functions. In XPCS, noise in the intensity correlation function becomes large when few photons are detected or the exposure time is short. An encoder–decoder model based on a convolutional neural network has been shown to extract the signal component from a noisy correlation function and to improve the accuracy of relaxation-time estimation [92]. This result raises the possibility of obtaining dynamics information even with short exposure times or low X-ray flux. Convolutional-network denoising has also been applied to coherent diffraction imaging, where it reduces the ambiguity of the reconstruction [93].

On the other hand, noise removal by neural networks may erase or distort dynamics that are not included in the training data. Anomalous relaxation processes or non-stationary changes that are present in only small numbers are particularly at risk of being removed as noise. It is therefore important to compare the data before and after processing and to validate the analysis results against known model data or independent measurements.

Guarding against this failure mode requires validation that is planned rather than incidental, and several practical measures are available. The first is validation on simulated data that contains deliberately inserted rare and transient events, verifying that these survive processing rather than being absorbed into the noise model. The second is to report processed and unprocessed results side by side, and to confirm that derived quantities such as autocorrelation decay constants and mean-square displacement slopes are unchanged within their uncertainties by the denoising step; a denoiser that alters the quantity of interest is not performing noise removal. The third is hold-out validation across independent samples and independent beamtimes rather than across frames of a single measurement, which are correlated and therefore give an optimistic estimate of generalization. The fourth is cross-validation against an independent technique, as in the comparison of EBMD with differential scanning calorimetry. The fifth is calibrated uncertainty quantification (UQ): it is not sufficient to report a confidence interval, since the interval must be shown to achieve its nominal coverage, and aleatoric and epistemic contributions should be separated [94,95]. Finally, non-stationarity should be treated explicitly. Autocorrelation analysis presupposes stationarity, so this should be tested rather than assumed, and where it fails, change-point detection is more appropriate than fitting a single decay constant to the whole record.

AI-assisted analysis has also been reported for XPCS, extracting the features of non-equilibrium dynamics from the data without assuming a specific correlation-function model in advance [96]. Such methods are effective for complex relaxation processes that single-exponential or stretched-exponential functions cannot represent adequately. They offer the possibility of extracting features associated with changes in the state of a material without limiting the description to model parameters alone.

In cryo-EM, cryoDRGN has been developed to analyze structural heterogeneity using a deep generative model [97]. This method not only classifies large numbers of particle images into a small number of discrete structural classes, but can also represent, within a low-dimensional space, the continuous structural changes that exist among particle images. It can therefore visualize the multiple structural states present in a sample and the continuous changes between them. The same generative approach has since been extended to cryo-electron sub-tomograms [98].

However, the continuous structural distribution obtained with cryoDRGN does not directly indicate the actual temporal order. Different structural states do not necessarily lie on a single continuous reaction pathway, and the rate of motion cannot be determined directly from the structural distribution. Interpreting the distribution of structural states as dynamics therefore requires additional information, such as time-resolved experiments, reaction models, and MD simulations.

Markov state models (MSMs) are used to extract metastable states and inter-state transition rates from large numbers of trajectories [99]. An MSM classifies structures or motions into multiple states and determines the probability of moving from one state to another after a fixed time. By integrating many short MD trajectories, it is possible to estimate slow state transitions that are difficult to observe in a single simulation, together with their time scales.

When an MSM is applied to particle trajectories obtained experimentally, the observation time, time resolution, trajectory length, and method of state classification strongly influence the results. If the trajectories are too short, or if unobservable states exist, the correct transition rates may not be obtainable. Verifying the Markovian property of the model and the validity of the state classification is therefore necessary.

Forward calculation is important for comparing experimental data and simulations quantitatively. The term refers to computing, from a model of the structure or motion, the signal that is actually observed in an experiment. For example, scattering intensities or correlation functions are computed from the atomic trajectories obtained by MD simulation and compared directly with the experimental data.

Combining the total scattering or diffuse scattering of protein crystals with MD simulation has been shown to allow analysis of the correlated motions among atoms within the crystal [100]. Whereas ordinary crystal structure analysis yields an average structure, diffuse scattering carries information on correlated deviations from that average structure. Comparing the scattering pattern computed from MD with the experimental results makes it possible to verify the validity of the atomic-motion model.

A method for computing the XPCS intensity correlation function from MD trajectories and comparing it with measured values has also been reported [101]. The method converts the time variation of the atomic coordinates into the intensity fluctuations observed in the experiment. It thus makes it possible to examine which molecular motions give rise to the relaxation times and correlation functions obtained experimentally.

Applying such forward calculations to DXT and DXB is an important task for the future. For DXT, the orientation of the labeling nanocrystal and the change in the position of the diffraction spot must be computed from the MD trajectory. These quantities are then compared with the motions in the θ and χ directions obtained experimentally. For DXB, the change in diffraction intensity at each detector pixel must be computed from the change in crystal orientation, and the resulting ACF and relaxation time compared with the experimental values.

Such analyzes must distinguish the motion of the target molecule itself from the motion of the linker and the gold nanocrystal. Moreover, different molecular motions may produce similar diffraction-spot displacements or intensity fluctuations. Evaluating the identifiability—that is, how uniquely the molecular motion can be inferred from the observed signal—therefore becomes important. By iterating experiment, forward calculation, and parameter estimation, a closed-loop analysis that links the observed data to the molecular model is expected to become possible.

The reliability of such forward calculations depends greatly on the accuracy of the simulation used. Conventional MD has employed empirical force fields, but their accuracy is limited. Ab initio (first-principles) molecular dynamics [102] provides high accuracy based on the electronic states, although the computational cost limits the systems and time scales that can be treated. Recently developed machine-learning interatomic potentials achieve accuracy close to that of first-principles calculations at a computational cost close to that of classical MD. High-dimensional representations based on neural networks [103] and deep potentials that scale them up to large systems [104] have been proposed. Furthermore, the latest models incorporating symmetry (equivariance) have been reported to achieve high transferability even from a small amount of training data [105]. High-accuracy force fields form the foundation of the forward calculations used for comparison with experiment.

Another essential challenge is the gap in time scales. Whereas ordinary MD reaches times on the order of nanoseconds to microseconds, the motions observed by DXT and DXB extend from microseconds to seconds. Enhanced sampling methods are used to bridge this difference [106]. They include metadynamics, which promotes exploration of the free-energy landscape [107], and replica-exchange molecular dynamics (REMD), which exchanges replicas at multiple temperatures to help escape from locally stable states [108]. In addition, coarse-grained MD [109], which represents groups of atoms together, makes it possible to treat larger systems and longer times. Combining these methods with approaches that reconstruct slow transitions from large numbers of short trajectories, such as the MSMs described above [99], makes the motions in the time domain observed experimentally tractable in simulation.

In DXT and DXB, the observable quantity is the change in orientation of the labeling gold nanocrystal itself. Forward calculation computes the orientational ACF or rotational diffusion coefficient of the target molecule (as well as of the label and linker) from the MD trajectory. This quantity is then converted into the decay constant of DXB or the angular-direction MSD of DXT. Methods for obtaining the rotational diffusion tensor and orientational relaxation time from MD trajectories have been established [110]. In addition, methods for evaluating the translational and rotational diffusion of arbitrarily shaped nanoparticles from MD have been reported [111]. Attempts to compute peptides labeled with gold nanocrystals by REMD and to reproduce the rotational and tilting motions observed by DXT were made early on [112]. Nevertheless, quantitatively reproducing the fluctuation observables obtained from blinking and tracking, and separating the motion of the target molecule from that of the label and linker, remains a central challenge.

The applications described so far all apply large-scale data analysis after the measurement has finished, in order to extract a physical quantity from a data set that has already been recorded. A distinct and more recent development is to close the loop during the experiment itself. Large-scale data analysis has begun to be used not only for post-measurement analysis but also for decision-making during measurement. At synchrotron radiation facilities, research is advancing on incorporating artificial intelligence and big-data analysis into the experimental system so that data are processed automatically immediately after acquisition [113]. If data quality, sample state, the occurrence of phase transitions, and similar features can be judged during measurement, the measurement conditions can be changed on the spot.

Furthermore, autonomous beamlines that automatically change the measurement position, temperature, exposure time, energy, and other parameters on the basis of the results of online analysis are also under development [114]. In such closed-loop measurements, measurement, analysis, and the determination of the next measurement conditions are repeated automatically. A major advantage is that, within limited beamtime, conditions that yield more information can be prioritized for measurement. Autonomous closed-loop campaigns have now been completed on real materials libraries [115], and machine-learning guidance has been implemented inside the electron microscope itself [116].

In the future, integrating simultaneous measurement of multiple sites by multiple labeling, inverse analysis using deep learning, time-resolved measurement under operando conditions, autonomous measurement, and large-scale MD simulation is expected to become important. At present, however, inverse analysis of DXT, DXB, SAXB, and TXB using deep learning is not yet sufficiently established. The creation of training data, the incorporation of physical laws, and the evaluation of uncertainty in the estimated results remain tasks for the future.

Computation and data analysis are not add-on processing performed after the experiment. They are the elements needed to convert the angles, intensities, correlation functions, particle trajectories, and other quantities obtained in time-resolved measurements into molecular motions and material properties. The data analysis and computational models must therefore be designed as part of the measurement system, alongside the source, the detector, and the sample environment.

Integrating measurements that introduce a time axis with large-scale data analysis makes it possible to evaluate not only the average structure, but also the temporal changes in structure, the heterogeneity of the dynamics, and the transitions between states. In future materials characterization, it will be important not to treat experiment and computation separately, but to integrate the two into a single analytical system.

## 8. Summary and Outlook

In this review, we have surveyed the ongoing extension of materials characterization using X-rays and electron beams from static analysis, which yields an average structure, toward dynamic analysis, which introduces a time axis. The development of high-brilliance sources and of fast, high-sensitivity detectors that have almost entirely eliminated readout noise has dramatically shortened exposure times. A wide time domain ranging from seconds to femtoseconds has thereby become measurable. Even among these dynamic measurements, however, the type of motion being measured (the observable) differs from method to method. The dynamics that a given method can capture cannot be represented by a single frame rate or pixel size; they are determined by the combination of the observable, the accessible time window, and the detectable spatial scale. Moreover, the time axis itself is constructed differently in each case, so the physical meaning denoted by the word “dynamics” also differs among methods. It may be the kinetics of an average structure reconstructed by using an induced reaction as a stroboscope of a synchronized ensemble, the relaxation time obtained from the correlation of spontaneous fluctuations, or the stochastic trajectory of a single object tracked in real time. Each method should therefore not be ranked by a single number, but understood in a complementary manner, once its observable, its type of motion, and the construction of its time axis have been made explicit.

Methods such as time-resolved X-ray crystallography, coherent X-ray scattering, neutron scattering, and time-resolved electron microscopy are powerful means of capturing reaction-associated structural changes as well as fluctuations, diffusion, and relaxation over a wide time domain. However, most of them average the signal from many molecules, unit cells, or irradiated regions. In contrast, DXT, DXB, SAXB, TXB, and EBMD use gold nanocrystals or gold nanoparticles as probes. They report motion through mutually distinct observables—angular change, intensity fluctuation, and real-space displacement—and in each case without passing through a global ensemble average: DXT and EBMD by tracking individual objects, and DXB, SAXB, and TXB by resolving the fluctuation statistics of a single pixel or illuminated volume. These approaches cover different time domains and spatial scales, and together they form a single family of methods for materials measurement.

The angular changes, intensity fluctuations, ACFs, MSDs, and particle trajectories returned by these measurements are not, in themselves, directly connected to molecular motion or material properties. Linking the observables to models requires a second pillar that stands on an equal footing with the measurement itself. That pillar comprises both the forward calculation, which computes the experimentally obtained signal from MD simulations, and the inverse problem (Bayesian inference and machine learning), which estimates structure and motion from the observations. The integration of DXT/DXB with MD has already been undertaken at the level of mechanistic interpretation. Rigorously forward-calculating the fluctuation observables obtained from blinking or tracking, and separating the motion of the target molecule from that of the label and linker, nevertheless remains a central challenge. Physics-informed machine learning and UQ provide a promising framework for addressing it.

In drawing these threads together, it is worth restating the distinction that runs through this review. DXT and EBMD follow individual objects and yield genuine single-object trajectories. DXB, SAXB and TXB derive their signal from many scatterers within a single pixel or illuminated volume; they replace a global ensemble average with a spatially localized statistical one, which preserves local heterogeneity that a bulk measurement would wash out, but they are not single-molecule measurements. Conflating the two classes overstates what the latter can deliver, and the value of these methods is better served by describing them accurately.

Taken together, the integration of measurements that introduce a time axis with large-scale data analysis and computational models is becoming the foundation of a new materials characterization. This characterization goes beyond the average structure to quantify the temporal evolution of structure, the heterogeneity of dynamics, and transitions between states. Looking ahead, researchers will need to design not only the source, detector, and sample environment but also the data analysis and the forward- and inverse-problem models as a single measurement system. Such integration of experiment and computation, rather than their separation, will be the key to establishing dynamic measurement at the single-molecule and single-particle scale in materials science.

## Figures and Tables

**Table 2:** Comparison of the nanoprobe-based time-resolved methods described in this section. The fifth column distinguishes methods that follow individual objects from those that return a spatially localized statistical average.

Method	Measured Observable	Accessible Time Range	Spatial Scale and Type of Motion	Single-Object vs. Localized Statistical	Instrumentation	Radiation-Damage Risk	Main Limitations and Representative Applications
**DXT**	Angular trajectory (tilting θ, twisting χ) of the Laue spot from one labelled nanocrystal [50,51]	Microsecond order; 100 µs per frame in the study shown in Figure 4 of this review [52]	Sub-mrad to tens of mrad; rotational (tilting and twisting) motion of the labelled site [50,51]	Single-object: one Laue spot corresponds to one labelled molecule [53]	White (Laue) X-rays at a synchrotron; 2D detector [50,51]	High; white (Laue) beam and prolonged tracking [50,51]; higher dose than DXB [54]	Requires a nanocrystal label and immobilization; label size affects absolute values (Section 5) [55]. Channels and receptors [52,56,57]; hydrogels [58]
**DXB**	Blinking of the diffraction intensity from many labelled nanocrystals, analysed pixel by pixel [54]	Milliseconds to several thousand seconds [59]; 890 ns at an XFEL [60]	Picometre-scale rotational displacement; rotational diffusion, statistically averaged [59]	Localized statistical: many contributors within one pixel [54]	Monochromatic X-rays; synchrotron, XFEL or laboratory Cu-anode source [59,60]	Lower than DXT; suited to long-duration measurement [54]	Yields no individual trajectories. Proteins with a laboratory source [59]; rubber and carbon black [60]; polymer thin films by GI-DXB [61]; molecular crystals [62]
**SAXB**	Intensity fluctuation in the small-angle scattering region, analysed pixel by pixel [63]	Δt = 70 µs per frame [63]	Nanometre to mesoscale; lamellae, domains and aggregates; fluctuation of domain spacing [63]	Localized statistical: all structures within the irradiated volume [63]	Synchrotron SAXS with a 2D photon-counting detector [63]	Moderate to high; the X-ray-induced change is itself measurable and is suppressed at 95 K [63]	Not a single-lamella measurement; interpretation requires a structural model [63]. Degradation of protein crystals [63]
**TXB**	Intensity fluctuation of the transmitted X-ray image, via a CsI scintillator and a high-speed camera [64]	900 ns exposure per frame; 10,000 frames; autocorrelation to a maximum lag of 2.25 ms [64]	Bulk specimen, 20 µm per pixel; sub-microsecond fluctuation inside the material [64]	Localized statistical: per pixel [64]	Laboratory X-ray source; CsI scintillator; high-speed camera [64]	Low; laboratory source [64]	No structural specificity; discrimination requires PCA and LDA [64]. Resins of near-identical absorption, PEEK vs. PEI [64]
**EBMD**	Real-space TEM trajectories and mean-square displacement of individual gold-nanoparticle tracers [65]	TEM video acquisition; frame interval set by the camera [65,66]	Nanometre to micrometre displacement; translational diffusion of the tracer [65,66]	Single-object: per nanoparticle [65]	TEM with a heating holder [65,66] or a liquid cell [67,68]	High; the diffusion mode itself changes with electron dose [69]	Vacuum or liquid-cell constraints; the beam may drive the observed motion [68,69]. Polymer phase transitions [65]; lipid membranes [66]

## Data Availability

No new data were created or analyzed in this study. Data sharing is not applicable to this article.

## Abbreviations

ACF	autocorrelation function
BCDI	Bragg coherent diffractive imaging
cryo-EM	cryogenic electron microscopy
DLSR	diffraction-limited storage ring
DXB	diffracted X-ray blinking
DXT	diffracted X-ray tracking
EBMD	electron-beam molecular dynamics
EXAFS	extended X-ray absorption fine structure
FEG	field emission gun
LDA	linear discriminant analysis
MBA	multi-bend achromat
MD	molecular dynamics
MSD	mean-square displacement
MSM	Markov state model
NSE	neutron spin echo
PCA	principal component analysis
PINN	physics-informed neural network
QENS	quasi-elastic neutron scattering
REMD	replica-exchange molecular dynamics
SAXB	small-angle X-ray blinking
SAXS	small-angle X-ray scattering
SFX	serial femtosecond crystallography
TR-SFX	time-resolved serial femtosecond crystallography
TXB	transmitted X-ray blinking
UQ	uncertainty quantification
XFEL	X-ray free-electron laser
XPCS	X-ray photon correlation spectroscopy
XSVS	X-ray speckle visibility spectroscopy
